# Nintedanib Mitigates Radiation-Induced Pulmonary Fibrosis by Suppressing Epithelial Cell Inflammatory Response and Inhibiting Fibroblast-to-Myofibroblast Transition

**DOI:** 10.7150/ijbs.92620

**Published:** 2024-06-11

**Authors:** Jingyao Tu, Xinyi Chen, Chunya Li, Chaofan Liu, Yongbiao Huang, Xi Wang, Hang Liang, Xianglin Yuan

**Affiliations:** 1 Department of Oncology, Tongji Hospital, Tongji Medical College, Huazhong University of Science and Technology, Wuhan, China.; 2 Department of Orthopedics, Union Hospital, Tongji Medical College, Huazhong University of Science and Technology, Wuhan, China.

**Keywords:** Radiation-induced pulmonary fibrosis, Nintedanib, Epithelial cells, Inflammatory response, Fibroblast-to-myofibroblast transition.

## Abstract

Radiation-induced pulmonary fibrosis (RIPF) represents a serious complication observed in individuals undergoing thoracic radiation therapy. Currently, effective interventions for RIPF are unavailable. Prior research has demonstrated that nintedanib, a Food and Drug Administration (FDA)-approved anti-fibrotic agent for idiopathic pulmonary fibrosis, exerts therapeutic effects on chronic fibrosing interstitial lung disease. This research aimed to investigate the anti-fibrotic influences of nintedanib on RIPF and reveal the fundamental mechanisms. To assess its therapeutic impact, a mouse model of RIPF was established. The process involved nintedanib administration at various time points, both prior to and following thoracic radiation. In the RIPF mouse model, an assessment was conducted on survival rates, body weight, computed tomography features, histological parameters, and changes in gene expression. *In vitro* experiments were performed to discover the mechanism underlying the therapeutic impact of nintedanib on RIPF. Treatment with nintedanib, administered either two days prior or four weeks after thoracic radiation, significantly alleviated lung pathological changes, suppressed collagen deposition, and improved the overall health status of the mice. Additionally, nintedanib demonstrated significant mitigation of radiation-induced inflammatory responses in epithelial cells by inhibiting the PI3K/AKT and MAPK signaling pathways. Furthermore, nintedanib substantially inhibited fibroblast-to-myofibroblast transition by suppressing the TGF-β/Smad and PI3K/AKT/mTOR signaling pathways. These findings suggest that nintedanib exerts preventive and therapeutic effects on RIPF by modulating multiple targets instead of a single anti-fibrotic pathway and encourage the further clinical trials to determine the efficacy of nintedanib in patients with RIPF.

## Introduction

Over 50% of patients diagnosed with thoracic malignancies require radiation therapy, a critical therapeutic strategy for various malignancies. However, thoracic radiotherapy can lead to radiation-induced lung injury (RILI). RILI is characterized by acute radiation pneumonitis and late radiation-induced pulmonary fibrosis (RIPF) 1-3. Apart from disrupting the successful completion of radiotherapy schedules, RIPF contributes to chronic lung dysfunction, thereby adversely impacting the quality of life of patients. It can also lead to pulmonary heart disease and, in severe cases, result in fatality 4, 5. Currently, effective preventive or therapeutic interventions are not available for RIPF. Therefore, a dire need exists to develop efficacious clinical therapeutic strategies for this condition.

The mechanism underlying the onset and progression of RIPF remains unclear. Research has indicated that the occurrence of RIPF is a dynamic response process influenced by multiple factors 6, 7. Specifically, radiation-induced epithelial cell damage and fibroblast activation play pivotal roles in mediating the development of RIPF 8, 9. The primary mechanisms initiating the process involve direct DNA damage and the generation of reactive oxygen species within epithelial cells 10, 11. Radiation stimulates the release of damage-associated molecular-pattern molecules from epithelial cells, leading to the recruitment of lymphocytes, macrophages and neutrophils 6, 12. The transmigration of immune cells promotes the release of various cytokines, such as interleukin (IL)-1β, IL-6, interferon (IFN)-γ, transforming growth factor (TGF)-β, and tumor necrosis factor (TNF)-α, which accumulate within the damaged lung tissue. These processes initiate an inflammatory response, which in turn leads to acute pneumonitis and the subsequent onset and progression of chronic pulmonary fibrosis through diverse signaling pathways 13, 14.

Radiation can also induce the activation and differentiation of fibroblasts, resulting in exaggerated and uncontrolled repair processes 15. The final step in the development of RIPF involves the fibroblast-to-myofibroblast transition 16. This transition is hallmarked by the expression of alpha-smooth muscle actin (α-SMA) and deposition of extracellular matrix (ECM) protein 17, 18. The excessive accumulation of myofibroblasts within the fibroblastic foci results in the contraction of lung tissue and an increased thickness of the alveolar-capillary membrane. This ultimately leads to an irreversible impairment in lung diffusing capacity, restrictive ventilatory insufficiency, and lung fibrosis 19, 20. Therefore, treatment with agents that alleviate epithelial cell damage and inhibit fibroblast-to-myofibroblast transition represents potential therapeutics for RIPF.

Drugs like corticosteroids or azathioprine are reported to be effective only against radiation pneumonitis. However, no effective therapeutic agents for RIPF have been identified ye 21, 22. Nintedanib is a triple tyrosine kinase inhibitor approved by the Food and Drug Administration (FDA). It explicitly targets FGFR1/2/3, PDGFRA/B, and VEGFR1/2/3 23. Additionally, it exerts effective therapeutic effects on idiopathic pulmonary fibrosis (IPF) by inhibiting disease progression 24, 25. Recent clinical trials have shown that nintedanib demonstrates strong therapeutic effects in the case of other chronic progressive fibrotic diseases, including coronavirus disease-triggered pulmonary fibrosis 26-28. Moreover, the preventive effects of nintedanib were compared to placebo in individuals with non-small cell lung cancer undergoing radiotherapy to assess their impact on radiation pneumonitis. The group treated with nintedanib exhibited a remarkably lower incidence of radiation pneumonitis-related events relative to the control group. However, the authors did not present conclusive evidence for the therapeutic effect of nintedanib on RIPF due to limitations in sample size and follow-up time 29.

These findings indicate that nintedanib serves as an effective treatment agent for IPF and other chronic progressive fibrotic diseases. However, its therapeutic impact concerning RIPF and the associated molecular mechanisms remains unelucidated. This study assessed the therapeutic efficacy of nintedanib utilizing murine models of RIPF *in vivo*. Additionally, the study focused on identifying therapeutic targets for nintedanib and underlying molecular mechanism in lung epithelial cells and fibroblasts. The regulatory effect of nintedanib on the cross-talk between epithelial cells and fibroblasts has also been investigated in this study. This research offers novel insights into the protection against radiation-induced lung tissue injury and establishes a foundation for the potential clinical application of nintedanib in RIPF treatment.

## Materials and Methods

### Reagents and animal experiments

Selleck Chemicals (Houston, TX, USA) supplied nintedanib. C57BL/6 mice (female; 6-8 weeks old; 18-20 g of body weight) were sourced from Vital River Laboratories (Beijing, China). They were kept in a specific pathogen-free environment. All procedures were carried out as per the International Guiding Principles for Animal Research guidelines and received approval from the Ethics Committee of Tongji Medical College, Huazhong University of Science and Technology (Wuhan, China).

Six groups of C57BL/6J mice were randomly assigned (n = 20/group): control group; nintedanib monotherapy group; radiation treatment (RT) group; Pre group, two days of pre-treatment with nintedanib and RT; 4wPost group, RT and nintedanib treatment at week 4 post-radiation; 12wPost group, RT and nintedanib treatment at week 12 post-radiation. For the radiation exposure, the mice were secured in a supine position with their limbs fixed on a plate. The single X-ray dose of 16 Gy was administered to the thorax, utilizing a clinical linear accelerator (6Mv X-rays, Elekta Precise, Stockholm, Sweden). The radiation was directed at the geometric center of the lung with a source distance of 100 cm and a dose rate of 600 cGy per minute. Nintedanib (30 mg/kg body weight, once daily for 4 weeks) was administered orally in the following way: beginning either two days before radiation (Pre group), at week 4 (4wPost group), or at week 12 (12wPost group). Mice in the control and RT groups received oral administration of saline. After radiotherapy, the survival of mice was assessed daily, and their body weight was monitored once every two weeks.

### Micro-computed tomography (CT) scan

Three mice were randomly selected from each group at weeks 4, 12, and 22 post-radiation and were then anesthetized with pentobarbital sodium. The thorax of mice was scanned using high-resolution micro-CT scanner (SkyScan 1176, Bruker, Billerica) at 50 kV, 500 μA, with an exposure duration of 265 ms. RadiAnt DICOM Viewer was utilized to reconstruct the images and quantify the lung tissue density in terms of Hounsfield units (HUs) commonly employed in clinical CT scanners.

### Lung histology and fibrosis scoring

At weeks 4, 12, and 22, the mice were euthanized through intraperitoneal injection of sodium pentobarbital (100 mg/kg body weight). The lung tissues were rapidly dissected and weighed. In the treatment groups, the lung weight was standardized to that of the control group. On the days set for CT examinations, three mice from each group were euthanized for histological analysis. First, overnight lung fixation was carried out in 4% formalin. Then, they were embedded in paraffin and sectioned into 5-μm-thick slices. To assess collagen deposition, these sections were treated with hematoxylin-eosin (H&E) and Masson's trichrome stains. Pulmonary fibrosis severity was gauged with the modified Ashcroft score 30, which varies from 0 to 8. Examination of five random microscopic fields at 200× magnification was conducted. The severity of fibrosis was ascertained by averaging the scores from all the fields examined.

### Immunohistochemical (IHC) analysis

Protein expression was analyzed via IHC using anti-αSMA (1:200, #19245, Cell Signaling Technology), anti-COL1A1 (1:100, #72026, Cell Signaling Technology), and anti-CTGF (1:200, A11456, ABclonal) antibodies following the guidelines of the manufacturer. To conduct the IHC scoring, five randomly chosen fields were examined under a light microscope. The IHC findings underwent a semi-quantitative analysis. Each sample received a score based on the intensity of the immunoreactive signal (strong = 3; moderate = 2; weak = 1; and negative = 0) and the percentage of cells staining positively (>50% positive cells = 3; 30-50% positive cells = 2; 10-30% positive cells = 1; and < 10% positive cells = 0). The total IHC score was computed by multiplying the two individual scores. Two pathologists independently assessed all slides using a light microscope. To prevent observer bias, all histological samples were assigned random numbers in a blinded manner.

### Cell lines and culture

Murine fibroblast cell line (L929 cells), human lung fibroblast cell line (MRC-5 cells), murine lung epithelial cell line (MLE-12 cells), and human lung epithelial cell line (BEAS-2B cells) were obtained from the cell bank of the Chinese Academy of Sciences (Shanghai, China). Dulbecco's modified Eagle's medium (Hyclone, Logan, UT, USA) was used for the cultivation of L929 and MLE-12 cells. Minimal essential medium (Hyclone, Logan, UT, USA) and Roswell Park Memorial Institute-1640 medium (Hyclone, Logan, UT, USA) were used for the cultivation of MRC-5 and BEAS-2B cells, respectively. Cells were cultured in complete medium with 10% fetal bovine serum (Gibco) at 5% CO_2_ and 37 °C. Cells at passages 3-5 were used for all experiments.

### Western blotting

To prepare the protein sample, lung tissues were homogenized. The extraction of cellular or tissue proteins was carried out using a radioimmunoprecipitation assay buffer (Beyotime, Shanghai, China) with phenylmethylsulfonyl fluoride and phosphatase inhibitors (Servicebio, Wuhan, China). Subsequently, the lysates were subjected to sodium dodecyl sulfate-polyacrylamide gel electrophoresis. The proteins, once separated, were moved to a polyvinylidene fluoride membrane (Millipore, Burlington, MA, USA). 5% bovine serum albumin was utilized to block the membrane for 1 hour. Then, it was incubated overnight at 4 °C with the primary antibodies specified below: anti-Smad2/3 (#8685), anti-p-Smad2/3 (#8828), anti-αSMA (#19245), anti-COL1A1 (#72026), anti-AKT (#4691), anti-mTOR (#2983), anti-p-mTOR (#5536), anti-extracellular signal-regulated kinase (ERK)1/2 (#4695), anti-p38 (#8690), anti-p-p38 (#4511), anti-p65 (#8242), anti-p-p65 (#3033), anti-COX-2 (#12282) (all 1:1000, Cell Signaling Technology) antibodies, anti-p-ERK1/2 (#4370), anti-p-AKT (#4060) (all 1:2000, Cell Signaling Technology) antibodies, anti-CTGF (A11456), anti-phosphoinositide 3-kinase (PI3K) (A4992), anti-p-PI3K (AP0427), anti-IL-1β (A16288), anti-IL-6 (A0286) (all 1:1000, ABclonal) antibodies, anti-TGF-β1 (1:1000, ab215715, Abcam), and anti-β-actin (1:5000, 60008-1-Ig, Proteintech) antibodies. Next, the membrane underwent incubation with the corresponding secondary antibodies (Promoter, Wuhan, China). The development of immunoreactive signals was assisted by SuperSignal West Pico PLUS Chemiluminescent Substrate (Thermo Scientific, Waltham, MA, USA). Visualization of these signals was achieved with the G: BOX Chemi X system (Syngene, Cambridge, UK). Analysis of blot images was conducted with ImageJ software.

### Co-culture assay

In the upper chamber, MLE-12 cells underwent overnight incubation. Subsequently, the MLE-12 cells received 8 Gy X-ray irradiation and immediately co-cultured with non-radiation-treated L929 cells for 48 hours (Figure 8A).

### Wound healing assay

After 24 hours of culture, the medium from the radiation-treated MLE-12 cells was collected and utilized as conditioned medium (CM) for subsequent experiments. The 6-well plates were used to place L929 cells, which were then treated with the CM of radiation-treated MLE-12 cells for 24 hours. Once the cell density reached about 75%-80% confluency, the monolayer was scratched with a sterile 200-µL pipette tip to generate a wound. Fresh phosphate-buffered saline (PBS) was then employed to wash the cells for the removal of the cell debris. Following a 24-hour incubation, three fields corresponding to each scratch wound were randomly selected and observed under microscopy to determine cell migration capability. The calculation of the ratio of the wound closure area to the original wound area was performed using ImageJ software.

### Transwell migration assay

In the upper chamber of transwell inserts, L929 cells were seeded and subjected to one of the following treatments: irradiation with 8 Gy X-ray or exposure to the CM obtained from radiation-treated MLE-12 cells. Following a 24-hour incubation in a standard incubator, non-migrating cells located on the upper side of the chamber filters were scraped by cotton swabs. Fixation was done with 4% paraformaldehyde, while 0.1% crystal violet dye stained the migrated cells on the lower membrane. Subsequently, stained cells were counted in three distinct fields within each insert. Duplicate experiments were conducted for each condition.

### Enzyme-linked immunosorbent assay (ELISA)

At week 22 post-radiation, the blood samples were obtained from the mouse eyeball. Those samples were left to clot at room temperature for 2 hours and subjected to centrifugation at approximately 1000 *g* for 20 minutes. The lung tissues were dissected, rinsed with ice-cold PBS to remove excess blood, and weighed before homogenization. Subsequently, the tissues were minced into small fragments and homogenized on ice in fresh lysis buffer using a glass homogenizer. The resulting homogenate underwent centrifugation at 10000 *g* for 5 minutes. After that, the supernatant was collected and immediately assayed. The expression levels of TGF-β1 (ELK1186) and IL-6 (ELK1157) in both the lung tissues and serum of RT mice were quantified utilizing ELISA. The commercial kits (ELK Biotechnology, Wuhan, China) were employed for these assays, and the guidelines of the manufacturer were strictly followed.

### RNA sequencing (RNA‑seq)

RNA-seq was conducted by Novogene (Beijing, China). Trizol (Takara Bio) was employed for total RNA extraction from the lung tissues (minimum of three samples per group), which then underwent purification for library preparation. Sequencing was carried out on an Illumina Hiseq platform. The analysis of differential gene expression among the four groups was conducted with the aid of the DESeq2 R package (version 1.20.0). Statistical significance for differential expression was defined as *P* < 0.05. The R 'clusterProfiler' package was utilized to conduct Gene Ontology (GO) and Kyoto Encyclopedia of Genes and Genomes (KEGG) pathway analyses on the differentially expressed genes (DEGs). Furthermore, the GO and KEGG datasets were independently assessed through Gene Set Enrichment Analysis (GSEA) with a GSEA tool (http://www.broadinstitute.org/gsea/index.jsp).

### Statistical analyses

All statistical analyses and graphing were executed utilizing GraphPad Prism 8. The data were presented as mean ± standard deviation. Comparison of means between two groups was performed utilizing Student's t-test, whereas one-way analysis of variance was employed for comparison among more than two groups. Statistical differences were deemed significant at *P* < 0.05 (^*^P < 0.05, ^**^P < 0.01, ^***^P < 0.001 and ****P < 0.0001).

## Results

### Impact of different treatments on mouse survival and health after thoracic radiation

**Figure 1:**
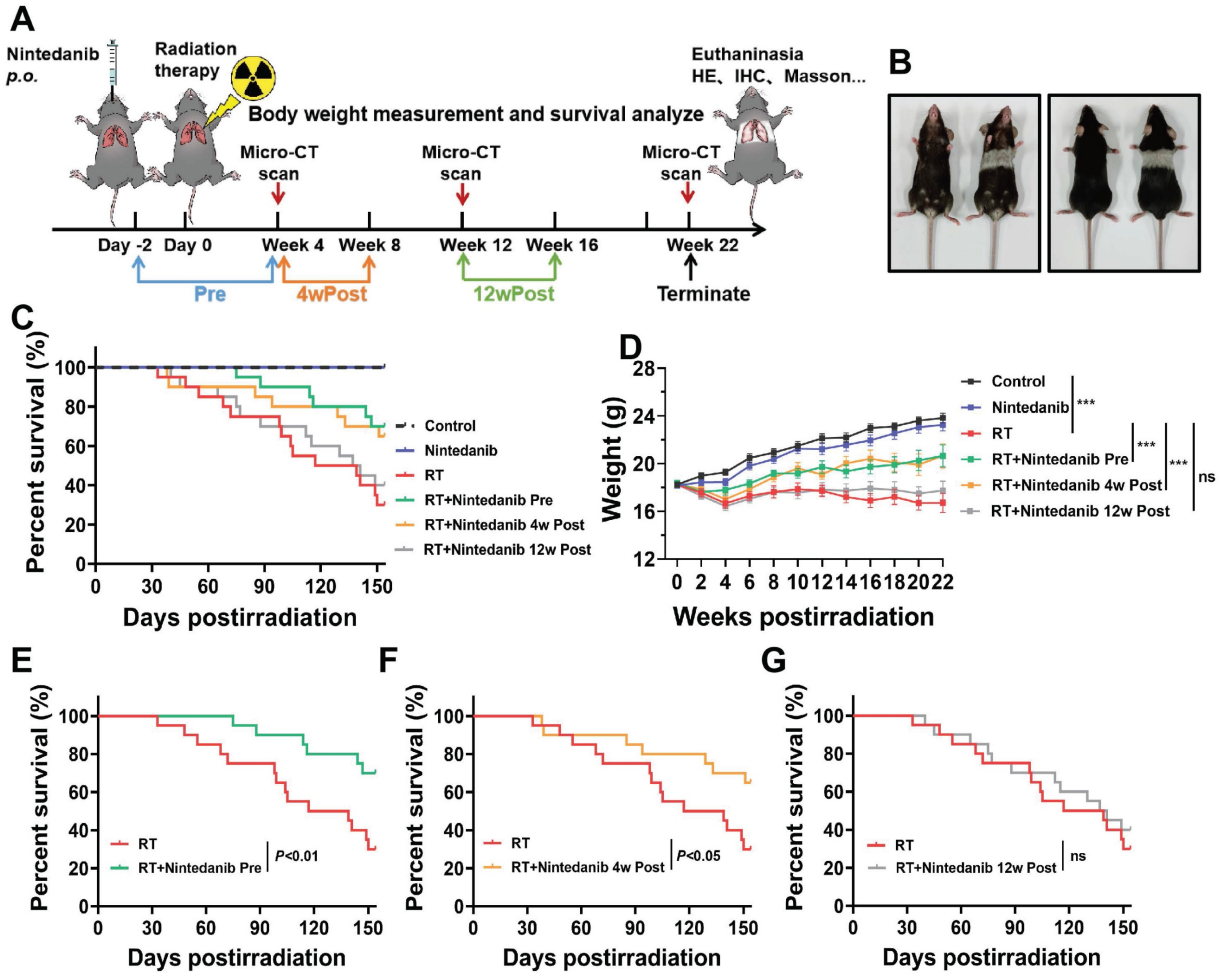
(A) Schematic representation of the study design illustrating the treatment regimen, timing of sacrifice, and parameters that were investigated. (B) Photograph of representative mice at week 22 post-radiation *vs.* control. (C) Kaplan-Meier analysis of the survival of mice subjected to thoracic radiation. (D) Body weight, a general health status indicator of mice. (E, F, and G) Kaplan-Meier survival curves based on nintedanib treatment.^ *^P < 0.05, ^**^P < 0.01, ^***^P < 0.001, ns: not significant.

As depicted in Figure 1A, the thorax of mice in the Pre, 4wPost, and 12wPost groups was irradiated with 16 Gy X-ray. Nintedanib at dose of 30 and 60 mg/kg were used in RIPF mice model. The results showed that nintedanib at dose of 30 and 60 mg/kg attenuated the radiation-induced increase in lung density and Ashcroft score. Although the dose of 60 mg/kg seemed to provide better efficacy, the difference was not statistically significant when compared to the 30 mg/kg dose. In terms of the principle of toxicity reduction and efficacy enhancement, nintedanib at dose of 30 mg/kg was used in further experiments (Figure S1). The animals were dissected at weeks 4, 12, and 22 post-radiation. Several parameters were analyzed to elucidate the therapeutic effects of nintedanib on RIPF. At week 22 post-radiation, the hair within the irradiated field of the mice exhibited a change in color, turning gray (Figure 1B). Thoracic radiation proved to be uniformly lethal, resulting in the demise of most mice after six-month post-radiation. However, the overall survival of mice in the Pre and 4wPost groups exhibited a prolonging effect in a schedule-dependent manner (Figure 1C). Nintedanib treatment beginning two days before and four weeks after radiation exhibited survival advantages when the data were analyzed from the start of radiation treatment (*P <* 0.05) (Figure 1E-F). However, the survival of mice did not increase in the 12wPost group (Figure 1G). Body weight serves as a crucial indicator for assessing health status. The body weight of RT mice was notably diminished compared to that of control mice, as depicted in Figure 1D (*P <* 0.001). However, radiation-induced weight loss was alleviated in the Pre and 4wPost groups (*P <* 0.001). These outcomes suggest that preventive and early treatment of nintedanib can improve the health status of mice and alleviate mortality associated with RILI.

### Nintedanib alleviates radiation-induced pathological manifestations in the lung

**Figure 2:**
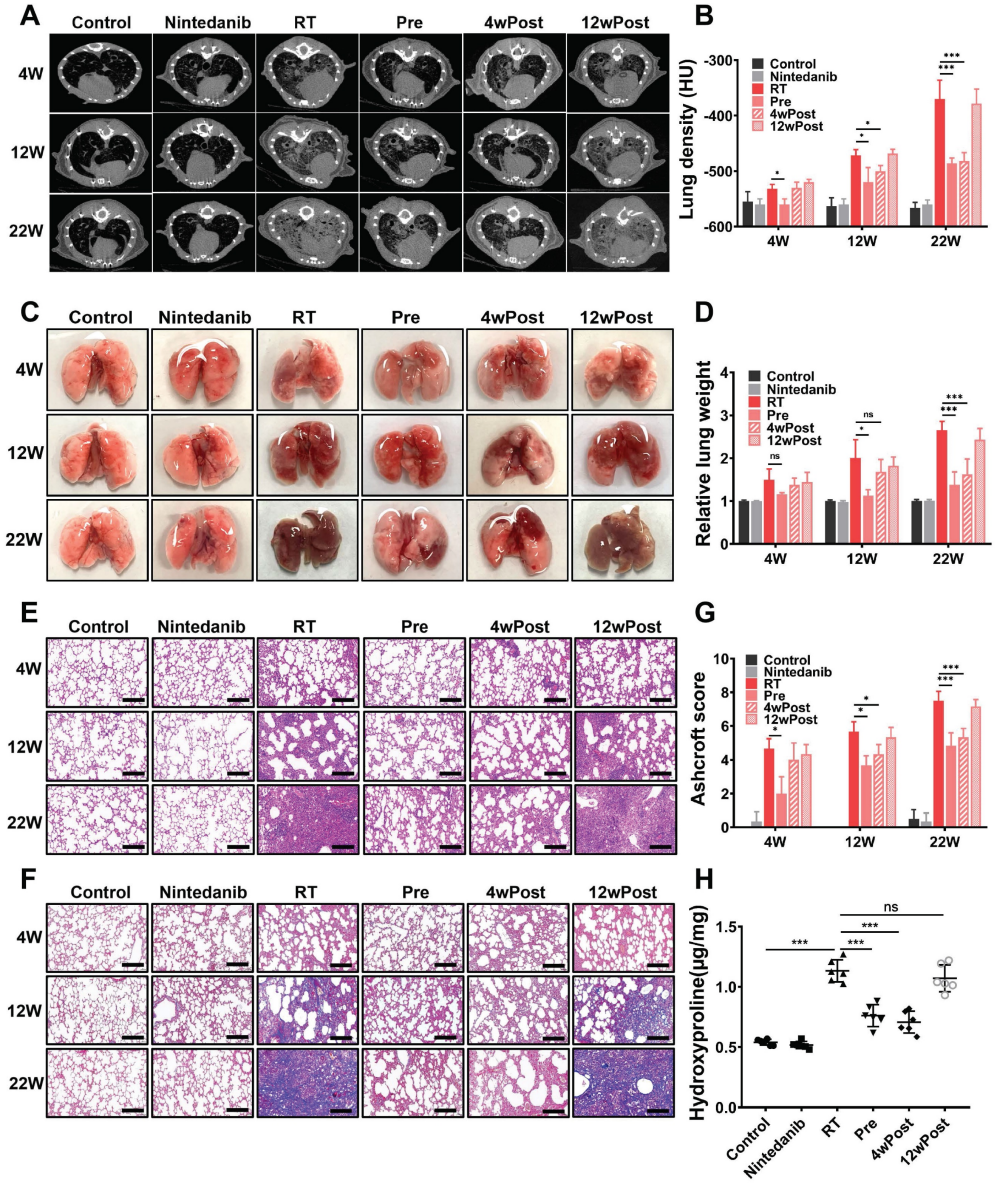
Effect of nintedanib on radiation-induced radiographic, morphological, and histopathological changes in the lungs of mice. (A) Representative micro-computed tomography (CT) images of different treatment groups at weeks 4, 12, and 22 post-radiation. (B) Assessment of lung density in micro-CT images. (C) Representative gross views of the lungs at weeks 4, 12, and 22 post-radiation. (D) Assessment of relative lung weight. (E) The results of hematoxylin and eosin (H&E) staining of lung samples obtained at weeks 4, 12, and 22 post-radiation (scale bar = 200 μm). (F) Masson's trichrome staining of lung samples from various groups at weeks 4, 12, and 22 post-radiation (scale bar = 200 μm). (G) Ashcroft score was used to determine the pulmonary fibrosis degree. (H) The hydroxyproline content in lung tissues of different groups was analyzed and quantified at week 22 post-radiation (n = 6 per group). The data are expressed as mean ± standard error of the mean. ^*^P < 0.05, ^**^P < 0.01, ^***^P < 0.001, ns: not significant.

An evaluation was performed to assess the factors underlying the pro-survival effects of nintedanib. An identifying feature of lung fibrosis is the increased tissue density, which can be analyzed through CT. The micro-CT images of mice depicted a significant increase in lung tissue density (opacity) from week 4 to week 22 post-radiation (Figure 2A-B). At week 4 post-radiation, CT scans suggested the presence of high-density foci distributed throughout the lungs in RT group, which is a typical manifestation of acute and subacute pneumonitis. Nintedanib pre-treatment attenuated the radiation-induced increase in lung density. At week 22, parenchymal opacity was markedly downregulated in the Pre and 4wPost groups compared with RT group, indicating decreased collagen deposition and improved lung aeration. However, the alterations in lung tissue density were not evident in the 12wPost group.

After radiation, the gross structural examination of the lung revealed a shrunken, scarred, and pale tissue with hard consistency, as well as multiple fibrous nodules in RT mice. In contrast, the lungs of mice from the Pre and 4wPost groups exhibited a physiological appearance characterized by increased elasticity and decreased fibrous nodules (Figure 2C). As shown in Figure 2D, the relative lung weight in the RT group was substantially greater than that of the control group (*P* < 0.05). However, early nintedanib treatment markedly mitigated the RT effect on relative lung weight, with weight approximately approaching the physiological weight (*P* < 0.05).

After radiation, H&E staining of lung tissues unveiled alveolar septal congestion and substantial accumulation of inflammatory cells in the alveolar space. However, H&E staining of esophagus or heart showed that thoracic irradiation at a dose of 16 Gy did not cause severe radiation esophagitis or radiation-induced cardiac toxicity n RIPF mice at week 22 post-radiation (Figure S2). Nintedanib alone did not lead to any pathological changes in lung architecture. In the Pre and 4wPost groups, the impact of radiation on the lung tissue was alleviated, demonstrated by a reduction in immune cell infiltration, decreased thickening of alveolar walls, and an increase in air spaces (Figure 2E). Collagen deposition in the pulmonary fibrotic foci, assessed by means of Masson trichrome staining (Figure 2F), was greater in RT mice compared to the control group. Meanwhile, collagen deposition in the Pre and 4wPost groups was less than that in the RT group. The Ashcroft score, which indicates the degree of interstitial fibrosis, was substantially low in the Pre group at weeks 4, 12, and 22 post-radiation. In the 4wPost group, the Ashcroft score was significantly lower at weeks 12 and 22 post-radiation (Figure 2G). However, pulmonary fibrosis was not alleviated in the 12wPost group at these time points. The results of hydroxyproline content analysis in lung tissues of different groups at week 22 post-radiation were consistent with the results of Ashcroft score (Figure 2H). These findings, consistent with the results of micro-CT scans, showed the anti-fibrotic potential of nintedanib at the early stage of RIPF.

**Figure 3:**
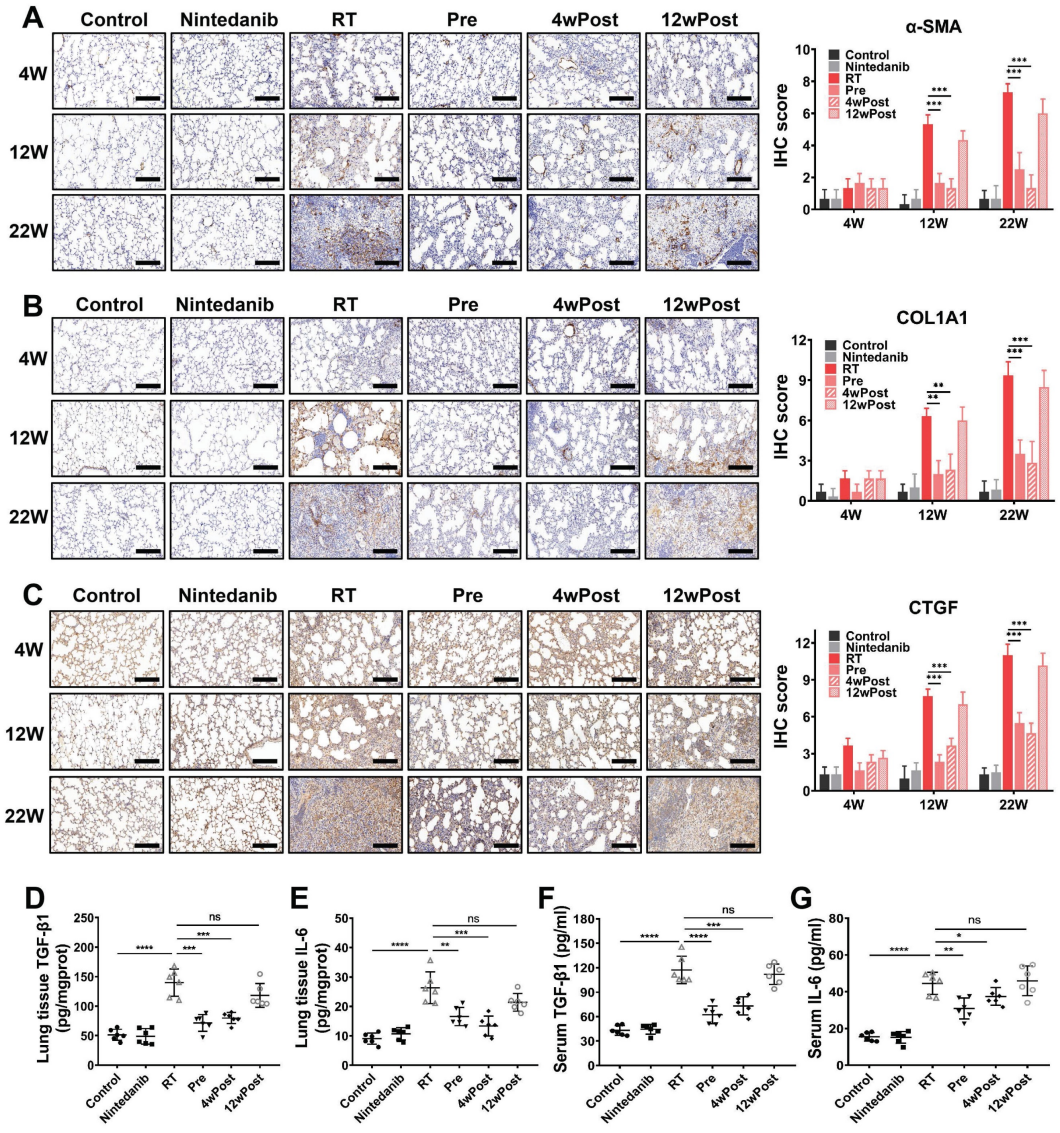
Effects of nintedanib on radiation-induced fibrotic response *in vivo*. The protein expressions of (A) α-SMA, (B) COL1A and (C) CTGF in mice lung tissue at 4, 12 and 22 weeks post-radiation were measured using IHC staining and semi-quantification with comparison to the control group. The levels of TGF-β1 and IL-6 in mice lung tissues (D, E) and serum (F, G) at 22 weeks post-radiation were measured by ELISA. Quantitative analysis of α-SMA, COL1A and CTGF were expressed as the means ± SEM. *P < 0.05, **P < 0.01, ***P < 0.001, ****P < 0.0001, ns: not significant. Scale bar = 200 μm.

For further assessment of the nintedanib effects on the fibrotic response, the expression levels of biomarkers (α-SMA, COL1A1 and CTGF) involved in pulmonary fibrosis development were analyzed through IHC staining. IHC staining for α-SMA (a biomarker of myofibroblast) revealed that nintedanib mitigated radiation-induced myofibroblast differentiation at weeks 12 and 22 post-radiation in the Pre and 4wPost groups (Figure 3A). Strong COL1A1 staining was found in the lung tissues of the RT group (*P* < 0.05). On the contrary, the pulmonary COL1A1-positive area in the Pre and 4wPost groups was lower than that in the RT group (*P* < 0.05) (Figure 3B). In the RT group, the expression levels of CTGF were elevated when compared to those in the control group (*P* < 0.05). Radiation-induced CTGF upregulation was mitigated in the Pre and 4wPost groups at weeks 12 and 22 post-radiation (*P* < 0.05) (Figure 3C). These are potential mechanisms underlying the anti-fibrotic effects of nintedanib, as myofibroblasts are involved in ECM deposition and lung remodeling during the development of lung fibrosis. As shown in Figure 3D-G, the levels of TGF-β1 and IL-6 in mice lung tissues and serum of RT mice were notably increased compared to that of control mice. However, radiation-induced increase in the levels of TGF-β1 and IL-6 were alleviated in the Pre and 4wPost groups. Interestingly, the alterations in either IHC staining (α-SMA, COL1A1 and CTGF) or ELISA analysis (TGF-β1 and IL-6) were not evident in the 12wPost group compared with RT group. This resulting data further suggested that nintedanib exhibits antifibrotic effects at the early stage of RIPF.

### Nintedanib inhibits the radiation-induced activation of inflammatory and fibrotic pathways

RNA-seq of lung tissues from the mice in Control, RT, Pre, and 4wPost groups was conducted to gain novel insights into the mechanisms that underlie the anti-fibrotic effects of nintedanib. DEG analysis demonstrated that 7573, 1996, and 1203 genes were differentially expressed in the RT, Pre, and 4wPost groups, respectively, compared to the control group. Heatmap of gene expression analysis in lung samples obtained at week 22 revealed that radiation significantly and coordinately altered the expression of several transcripts.

**Figure 4:**
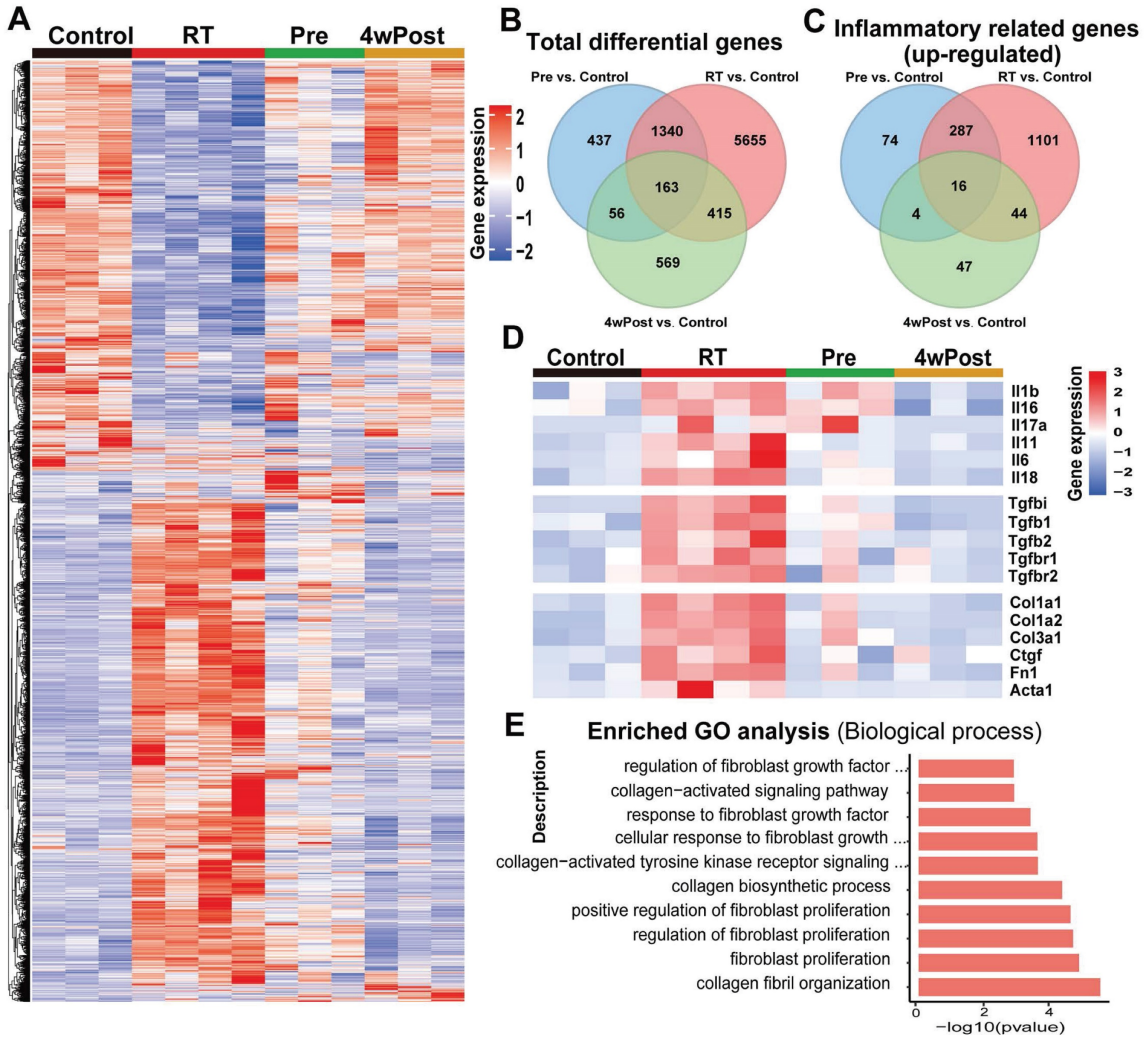
RNA sequencing (RNA-seq) analysis of pulmonary changes in different treatment groups. (A) Heatmap of significant differentially expressed genes (DEGs) (*P* < 0.05). (B) Venn diagram of total DEGs. (C) Venn diagram of DEGs enriched in the top 100 inflammatory-related Gene Ontology (GO) terms. (D) The expression levels of representative inflammation-related and fibrosis-related genes. (E) The top 10 substantially enriched fibrosis-related GO terms (radiation treatment (RT) group vs. control group).

Radiation-induced alterations in gene expression were mitigated in the Pre and 4wPost groups (Figures 4A and S3A). The Venn diagram provided an intuitive representation of the total number of DEGs (Figure 4B). The analysis of the DEGs enriched in the top 100 inflammatory-related GO terms revealed many upregulated inflammatory genes in the RT group. In contrast, the Pre and 4wPost groups exhibited fewer upregulated inflammatory genes (Figure 4C). Detailed inflammation-related GO terms are shown in Table S1. Individual genes analysis revealed that radiation upregulated the genes related to the inflammatory response and ECM remodeling, whereas nintedanib coordinately downregulated these genes (Figures 4D and S3B). RNA-seq data were subjected to GO analysis. The top 10 significant fibrosis-related GO terms enriched in the DEGs between RT and control groups are depicted in Figure 4E. The biological processes associated with pulmonary fibrosis (including collagen fibril organization and fibroblast proliferation) in the RT groups were upregulated in comparison to those in the control group. These findings indicate that the early treatment of nintedanib can alleviate inflammatory response and ECM remodeling in RT lung tissues at the transcriptomic level.

### Nintedanib ameliorates RIPF by suppressing the PI3K-AKT, MAPK and NF-κB signaling pathways

**Figure 5:**
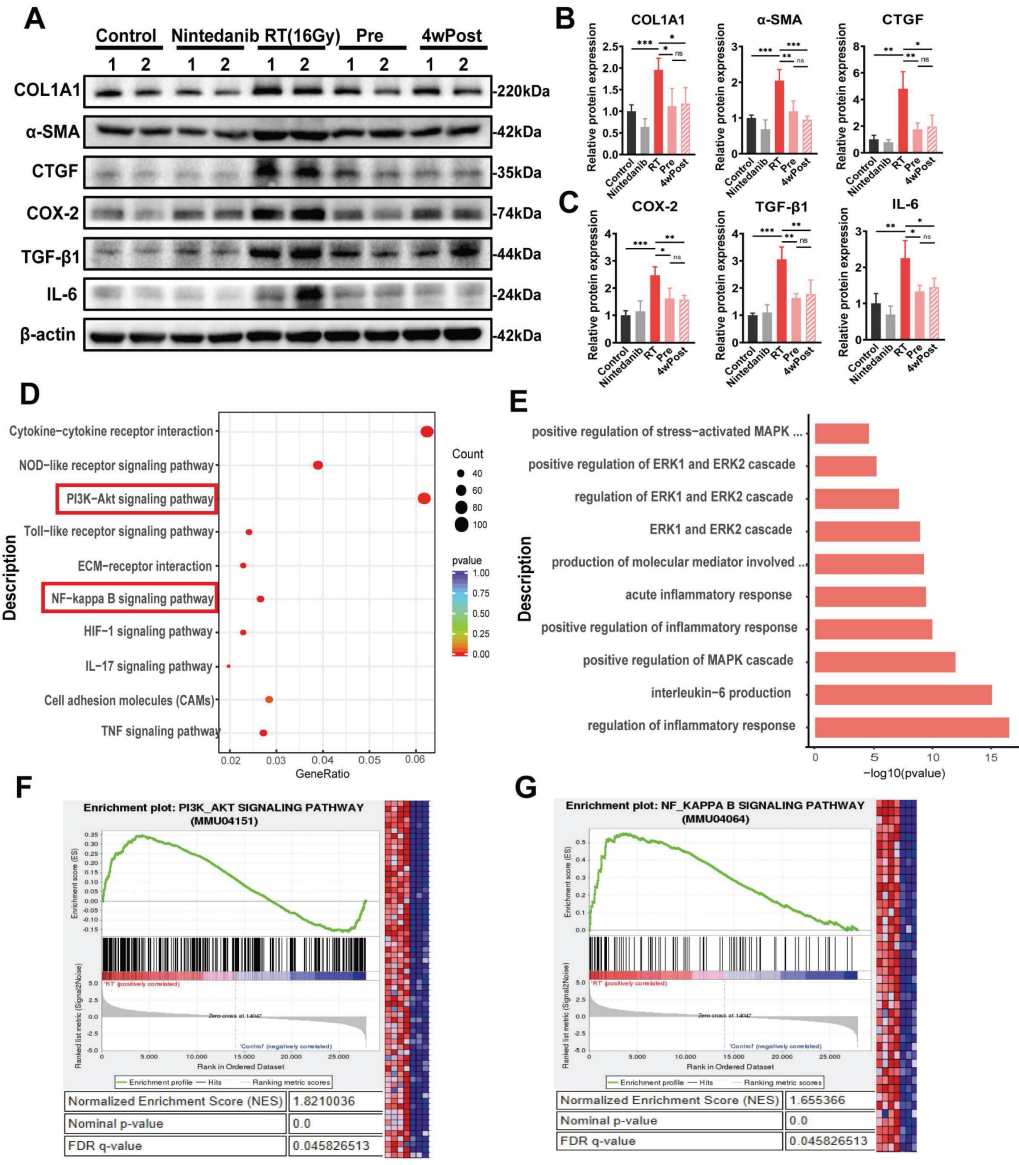
Analysis of the mechanisms responsible for the anti-fibrotic effects of nintedanib. (A) Representative western blotting analysis results of COL1A1, α-SMA, CTGF, COX-2, TGF-β1 and IL-6 in lung tissue of mice. Quantitative statistical results of (B) fibrosis-related and (C) inflammatory-related protein expression levels. (D) Dot plot of Kyoto Encyclopedia of Genes and Genomes (KEGG) analysis of differentially expressed genes (DEGs) between the radiation treatment (RT) and control groups. (E) Inflammation-related Gene Ontology (GO) terms in which the DEGs between the RT and control groups are enriched. (F and G) GSEA plots for RNA-seq data in the RT group compared to the control group. Normalized enrichment score (NES), nominal p-value and FDR q-value are stated on the plots. *P < 0.05, **P < 0.01, ***P < 0.001, ****P < 0.0001, ns: not significant.

Radiation upregulated the pulmonary levels of COL1A1, α-SMA, CTGF, COX-2, TGF-β1 and IL-6, which are associated with fibrosis and inflammation, in mice (n = 4/group) (Figures 5A-C and S4A). The KEGG and GO analyses were conducted to perform functional classification and enrichment of the RNA-seq data. KEGG analysis of the DEGs between the RT and control groups revealed the upregulation of signaling pathways associated with inflammatory response and ECM remodeling (PI3K-AKT and NF-κB signaling pathways) (Figure 5D). However, the activation of these pathways induced by radiation was suppressed in the Pre and 4wPost groups (Figure S4B). Additionally, the GO terms in which the DEGs between the RT and control groups were enriched are depicted in Figure 5E.

The MAPK signaling pathways were enriched in the Pre and 4wPost groups. Furthermore, gene set enrichment analysis (GSEA) showed that differentially expressed genes in the RT group were significantly enriched in “PI3K-AKT signaling pathways” (Figure 5F) and “NF-κB signaling pathways” (Figure 5G). As per the screening and experimental results, it was hypothesized that nintedanib alleviates the inflammatory response and ECM remodeling by acting on multiple targets rather than a single anti-fibrotic pathway.

### Nintedanib suppresses radiation-induced inflammatory response in epithelial cells through the PI3K/AKT and MAPK signaling pathways

**Figure 6:**
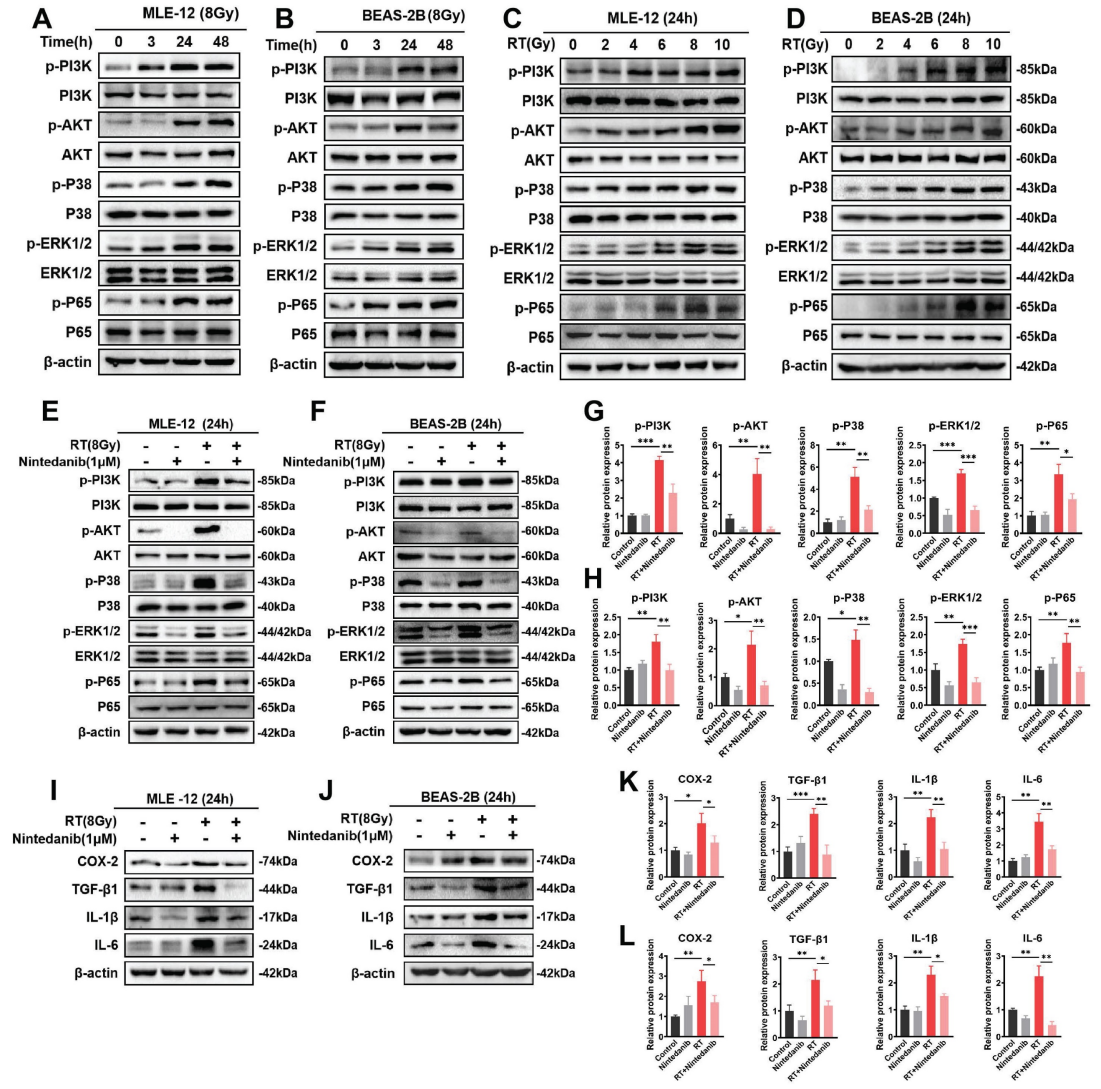
Nintedanib inhibited the radiation-induced activation of the PI3K/AKT and MAPK signaling pathways and inflammatory response in epithelial cells. (A-D) The time and dose of radiation were optimized by analyzing the protein levels using western blotting. Representative results of western blotting analysis of p-PI3K, PI3K, p-AKT, AKT, p-p38, p38, p-ERK1/2, ERK1/2, p-p65 and p65 in MEL-12 cells (E) and BEAS-2B cells (F) in different treatment groups, as well their corresponding quantitative statistical results in MEL-12 cells (G) and BEAS-2B cells (H). Representative results of western blotting analysis of COX-2, TGF-β1, IL-1β and IL-6 in MEL-12 cells (I) and BEAS-2B cells (J) in different treatment groups, as well their corresponding quantitative statistical results in MEL-12 cells (K) and BEAS-2B cells (L). ^*^P < 0.05, ^**^P < 0.01, and ^***^P < 0.001.

Multiple investigations have proposed an association between the inflammatory response in epithelial cells and various signaling pathways, including PI3K/AKT kinase, ERKs, and p38-MAPK 31-34. The findings from RNA-seq enrichment and experimental analysis have demonstrated the significant involvement of the PI3K/AKT and MAPK pathways in RIPF. To elucidate the molecular mechanism responsible for the therapeutic impact of nintedanib on RT epithelial cells, the expression levels of proteins associated with the PI3K/AKT and MAPK pathways were assessed through western blotting (Figure 6). The time and radiation dosage were optimized through a series of experiments. The phosphorylation of the majority of proteins related to PI3K/AKT and MAPK peaked in MLE-12 (Figure 6A and C) and BEAS-2B (Figure 6B and D) cells when irradiated with 8 Gy X-ray for 24 h. When the radiation dosage exceeded 8 Gy or the duration exceeded 24 hours, the expression of these proteins did not further increase. In addition, the expression of some proteins decreased, which may be due to more MLE-12 cells and BEAS-2B cells dying under 10 Gy radiation. Thus, the time and dosage were eventually determined to be 24 hours and 8 Gy. Nintedanib mitigated the radiation-induced upregulation of p-PI3K and p-AKT levels in MLE-12 cells and BEAS-2B cells. Furthermore, treatment with 1 μM nintedanib significantly mitigated the radiation-induced upregulation of p-p38 and p-ERK1/2. Moreover, nintedanib treatment markedly suppressed the radiation-induced upregulation of p65 (Figure 6E-H). Subsequently, the levels of COX-2, TGF-β1, IL-1β and IL-6 were examined to assess the impact of nintedanib on the radiation-induced inflammatory response of MLE-12 and BEAS-2B cells. In the RT group, the levels of COX-2, TGF-β1, IL-1β and IL-6 were significantly elevated compared to the control group (*P* < 0.05). However, pre-treatment with nintedanib before radiation markedly decreased their levels and the levels of their human homologs after radiation for 24 hours (*P* < 0.05) (Figure 6I-L). Thus, nintedanib effectively suppressed the radiation-induced upregulation of inflammatory factors in epithelial cells by regulating the signaling pathways, including PI3K/AKT and MAPK pathways.

### Nintedanib suppresses radiation-induced fibroblast-to-myofibroblast transition by the TGF-β/Smad and PI3K/AKT/mTOR signaling pathways

**Figure 7:**
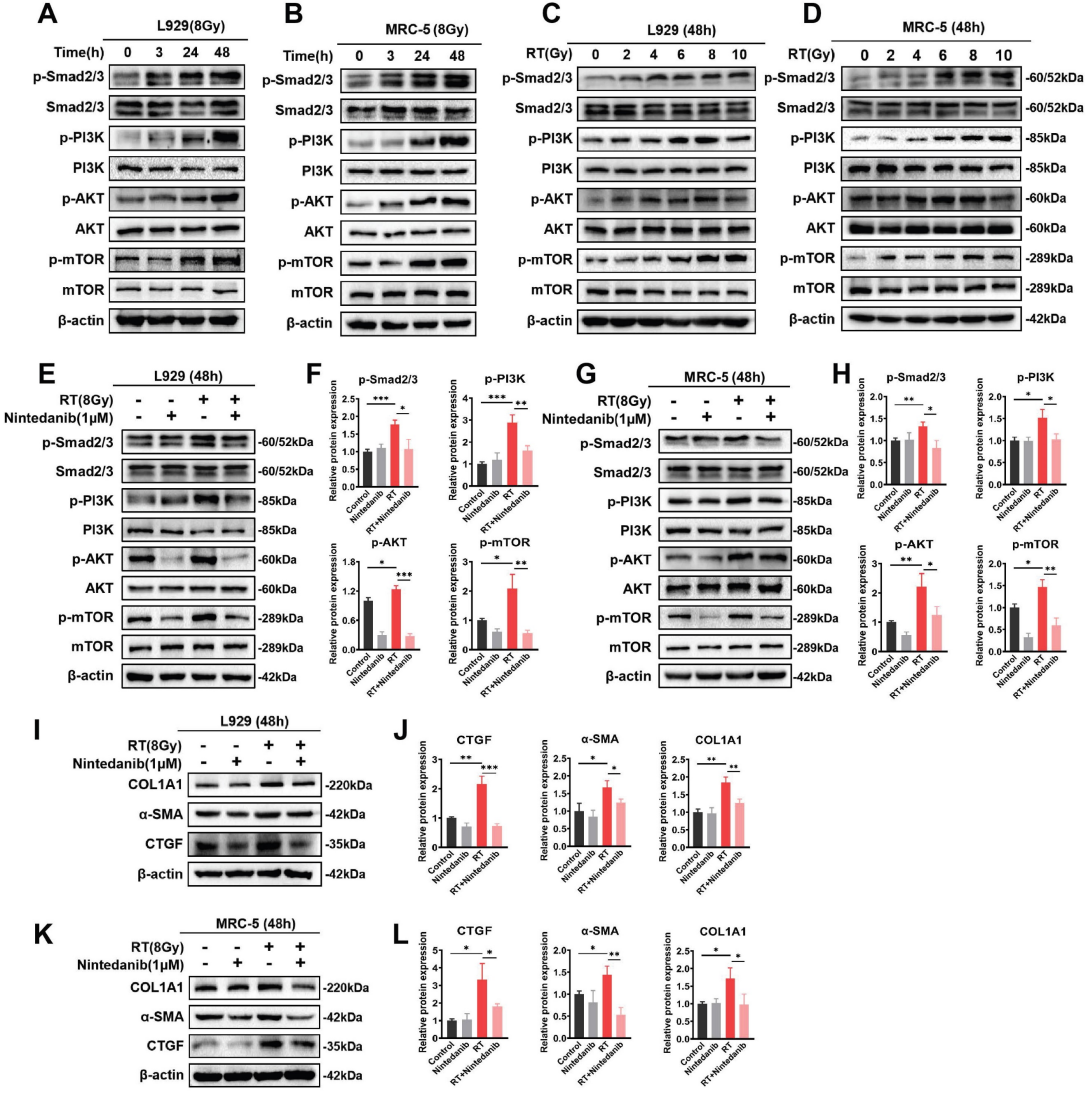
Nintedanib inhibited the radiation-induced activation of signaling pathways, including TGF-β/Smad and PI3K-AKT-mTOR, and the upregulation of fibrosis-related proteins in pulmonary fibroblasts. (A-D) The time and dose of radiation were optimized by evaluating protein levels using western blotting. Representative western blotting analysis results of p-Smad2/3, Smad2/3, p-PI3K, PI3K, p-AKT, AKT, p-mTOR and mTOR, in L929 cells (E) and MRC-5 cells (G) in different treatment groups, as well as their corresponding quantitative statistical results of protein levels in L929 cells (F) and MRC-5 cells (H). Representative western blotting analysis results of COL1A1, α-SMA and CTGF in L929 cells (I) and MRC-5 cells (K) in different treatment groups, as well as their corresponding quantitative statistical results of protein levels in L929 cells (J) and MRC-5 cells (L). ^*^P < 0.05, ^**^P < 0.01, and ^***^P < 0.001.

The TGF-β/Smad and PI3K/AKT/mTOR signaling pathways are critically involved in the formation of myofibroblasts during pathological processes associated with fibrosis 35-38. This study performed exploratory experiments to examine the nintedanib-mediated regulatory impact on the signaling pathway in RT pulmonary fibroblasts on the basis of RNA-seq results. The TGF-β/Smad and PI3K/AKT/mTOR signaling pathways exhibited the most significant variations. Thus, the phosphorylated Smad2/3, PI3K, AKT and mTOR were determined (Figure 7). The radiation time and dose were optimized by performing a series of experiments. The phosphorylation levels of the majority of proteins linked to the TGF-β/Smad and PI3K/AKT/mTOR signaling pathway peaked after 48 hours of exposure to 8 Gy X-ray in L929 (Figure 7A and C) and MRC-5 cells (Figure 7B and D). In the RT group, the phosphorylation levels of Smad2/3, PI3K, AKT and mTOR were elevated in comparison to those in the control group. Nintedanib treatment led to a reduction in the phosphorylation levels of Smad2/3, PI3K, AKT and mTOR (Figure 7E-H). To assess the inhibitory impact of nintedanib on the radiation-treated pulmonary fibroblasts to myofibroblasts transition, the morphological changes in L929 cells and the expression levels of COL1A1, α-SMA and CTGF in L929 cells and MRC-5 cells were analyzed through western blotting. Radiation-treated L929 cells appeared enlarged with stretched pseudopods. The morphology of L929 cells in the nintedanib-treated group closely resembled that of the control group (Figure S5). Western blotting highlighted that radiation elevated the protein levels of COL1A1, α-SMA and CTGF in L929 cells and MRC-5 cells. Nintedanib treatment downregulated the expression levels of COL1A1, α-SMA and CTGF at 48 hours post-radiation (Figure 7I-L). These findings suggest that nintedanib inhibits radiation-induced fibroblast-to-myofibroblast transition by regulating signaling pathways such as TGF-β/Smad and PI3K/AKT/mTOR pathways.

### Nintedanib inhibits fibroblast-to-myofibroblast transition induced by radiation-treated epithelial cells

**Figure 8:**
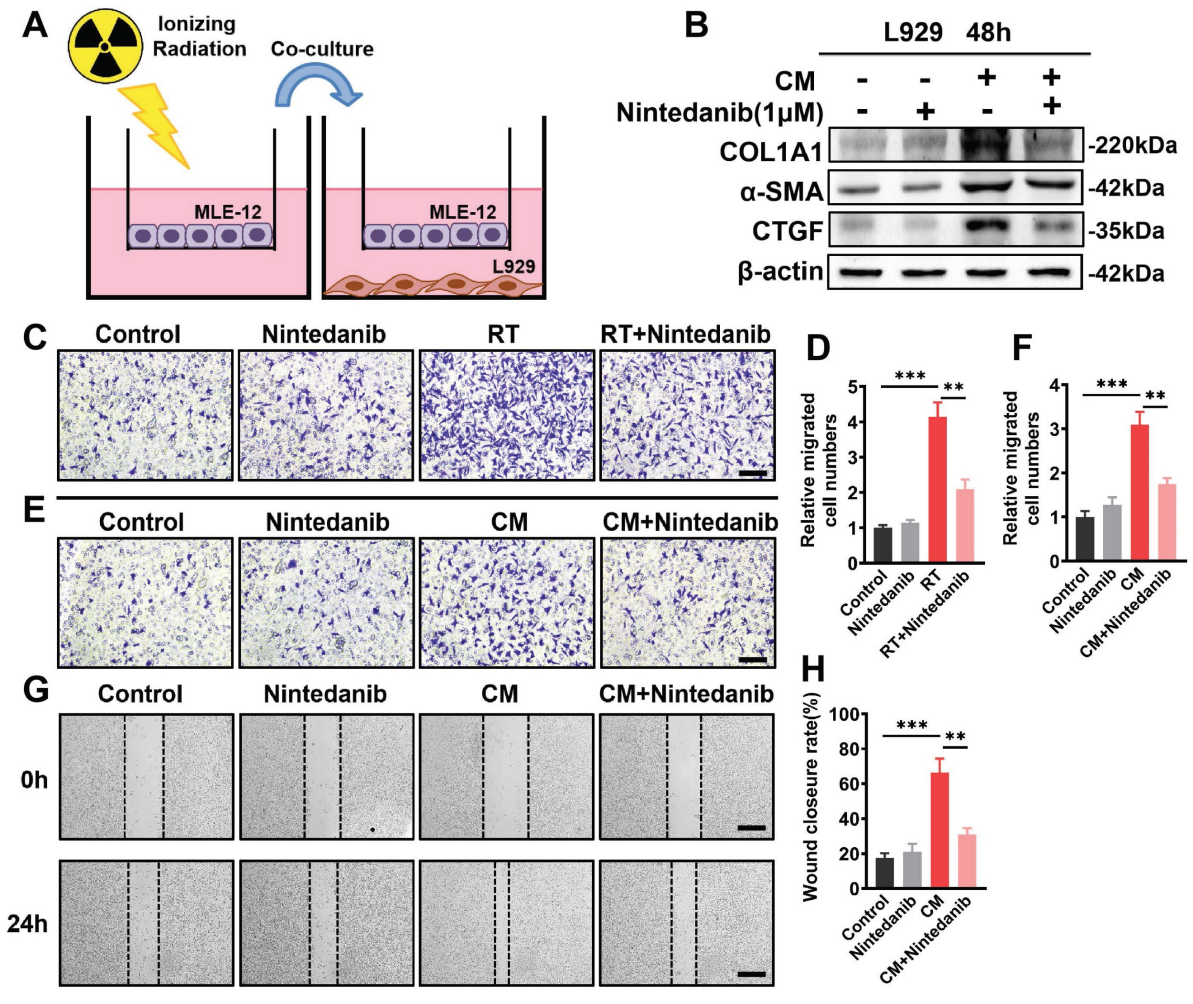
Nintedanib effects on radiation-mediated cross-talk between epithelial cells and fibroblasts. (A) Schematic representation of the co-culture setup: MLE-12 cells were placed in the upper chamber and subjected to overnight culture. L929 cells were seeded in the lower chamber. Subsequently, the MLE-12 cells were exposed to 8 Gy X-ray irradiation and immediately co-cultured with non-radiation-treated L929 cells for 48 hours with or without nintedanib (1 μM) treatment. (B) Representative protein bands of COL1A1, α-SMA and CTGF in L929 cells after co-culture for 48 hours. (C, D) Transwell assay and quantitative analysis showed the migration of L929 cells that irradiated with 8 Gy X-ray, with or without treatment of 1 μM nintedanib (scale bar = 100 μm). (E and F) Transwell assay, along with quantitative analysis, exhibited the migration of L929 cells after incubation in CM for 24 hours with or without treatment of 1 μM nintedanib (scale bar = 100 μm). (G and H) Wound closure assay for the assessment migratory capacities of L929 cells after incubation in CM for 24 hours with or without treatment of 1 μM nintedanib (scale bar = 200 μm). ^*^P < 0.05, ^**^P < 0.01, and ^***^P < 0.001.

The co-culture system (Figure 8A) was employed to examine the impact of nintedanib on the transition of fibroblast into myofibroblast induced by radiation-treated epithelial cells. Western blotting revealed that co-culturing with radiation-treated epithelial cells led to upregulation of the expression of the myofibroblastic markers, including COL1A1, α-SMA and CTGF, in L929 cells. However, nintedanib reversed the altered expression of these myofibroblast markers induced by radiation-treated MLE-12 cells (Figure 8B). Transwell system and scratch wound assay were employed to further examine the impact of nintedanib on the migration of fibroblasts induced by radiation-treated epithelial cells. The findings from the transwell assay depicted that radiation markedly promoted the migration of L929 cells, suggesting the transition of radiation-treated L929 cells into myofibroblast. Treatment with nintedanib mitigated the pro-migratory effect of radiation (Figure 8C-D). To examine the inhibitory effects of nintedanib on fibroblast-to-myofibroblast transition induced by radiation-treated epithelial cells, the medium of radiation-treated MLE-12 cells cultured for 24 hours was collected and utilized as conditioned medium (CM).

The results showed that the migration of L929 cells increased after incubation in CM for 24 hours, whereas nintedanib decreased the number of migrating cells (Figure 8E-F). The migration inhibitory ability of nintedanib was further confirmed using the scratch wound assay (Figure 8G-H), a widely used method for evaluating the migration of cells. As shown in Figure 8G, treatment with CM for 24 hours significantly increased the migration of L929 cells, whereas nintedanib suppressed the migration of L929 cells. The resulting data suggest that radiation-treated epithelial cells promote fibroblast-to-myofibroblast transition, and nintedanib may exert inhibitory effects on this process.

## Discussion

The pathogenesis of RIPF is hypothesized to be mediated by the release of various cytokines and growth factors, which include IL-6, TGF-β1, TNF-α, vascular endothelial growth factor, and platelet-derived growth factor 39, 40. The radiation-induced release of these cytokines and growth factors leads to an inflammatory response, which is clinically presented as subacute radiation pneumonitis. These signaling molecules activate fibroblasts and myofibroblasts in the long term. This activation causes fibrin deposition, resulting in permanent structural and functional alterations in the lung, ultimately leading to pulmonary fibrosis development 41-43.

**Figure 9:**
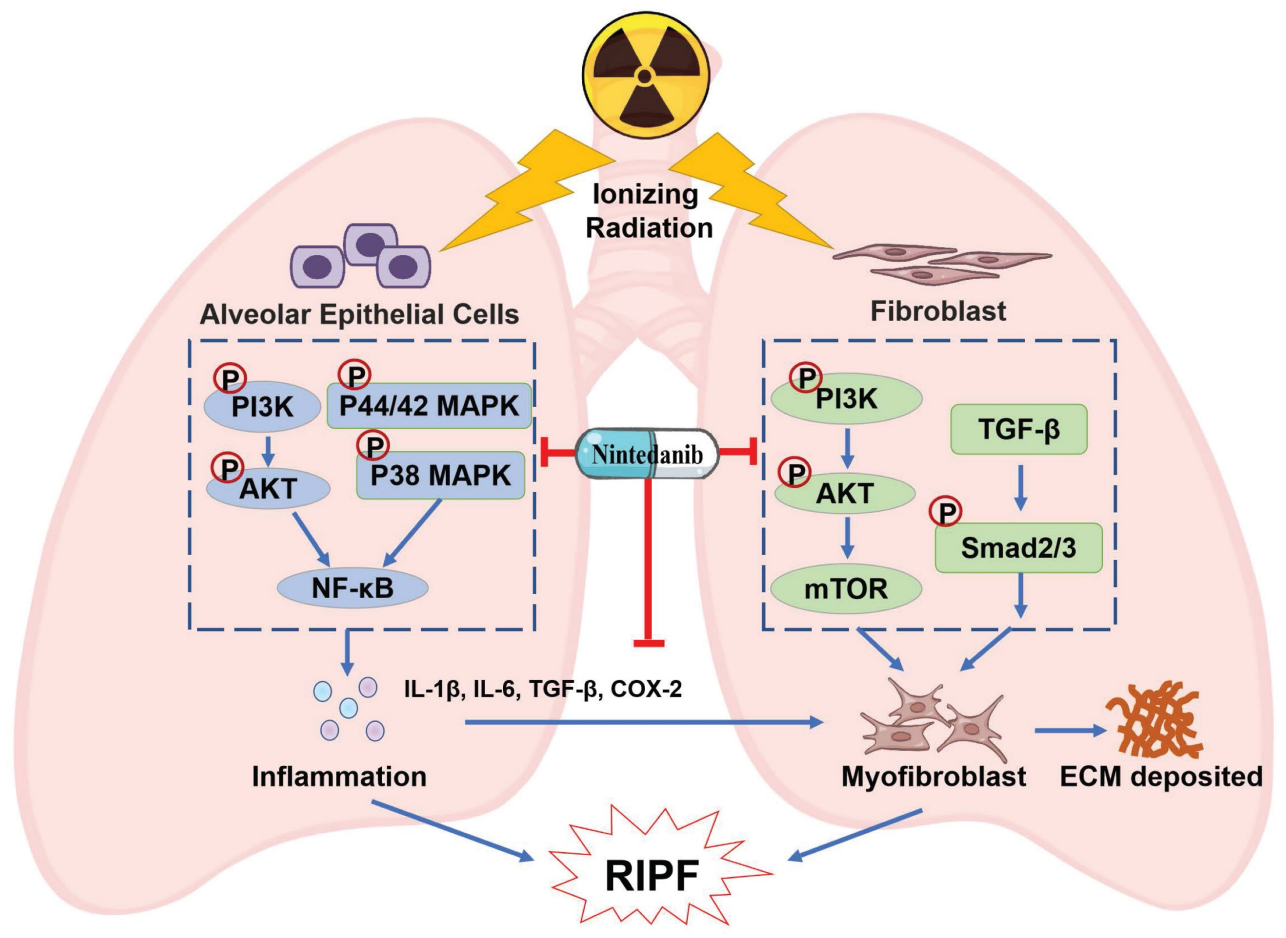
Schematic illustration of nintedanib effects in the RIPF.

Currently employed therapeutics have shown limited efficacy in alleviating RIPF 44-46. Thus, there is an urgent need to develop improved evidence-based management strategies for RIPF. Preclinical research has reported that nintedanib exhibits anti-fibrotic and anti-inflammatory effects in mouse models of lung fibrosis induced by bleomycin and silica 47. In clinical trials, nintedanib was observed to suppress the forced vital capacity (FVC) decline, thereby delaying IPF progression 48-50. Its approval has been extended to include chronic fibrosing interstitial lung diseases (ILDs) with a progressive phenotype and systemic sclerosis-associated ILD. This expansion was based on the observed benefits of nintedanib in delaying the FVC decline in the INBUILD and SENCIS trials 51, 52. Moreover, the addition of nintedanib to a prednisone taper has been observed to alleviate radiation pneumonitis 53. The combination therapy of nintedanib and dexamethasone resulted in a temporary improvement in RILI 54. However, these studies primarily investigated the efficacy of combining glucocorticoids with nintedanib for treating RILI, and the sample sizes are limited. The effect of monotherapy of nintedanib on RIPF and the underlying mechanism are unknown. Given the absence of well-established clinical treatment options for RIPF and the presence of overlapping pathways in both RIPF and IPF pathogenesis, this study aimed to assess the therapeutic potential of nintedanib for treating RIPF. This study yielded the following results: A) Nintedanib inhibited the development and progression of RIPF in mice. B) Nintedanib reduced the inflammatory response in epithelial cells by modulating the PI3K/AKT and MAPK signaling pathways. C) Nintedanib suppressed pulmonary fibroblast differentiation by regulating TGF-β/Smad and PI3K/AKT/mTOR signaling pathways. D) Nintedanib exerted regulatory effects on factors involved in the cross-talk between epithelial cells and fibroblasts during RIPF progression (Figure 9).

The administration of nintedanib attenuated radiation-induced lung remodeling in mice and effectively suppressed the previously initiated process of lung remodeling. The benefits were observed in both the Pre and 4wPost groups. Specifically, histological and longitudinal CT analyses demonstrated the prevention or attenuation of radiation-induced lung remodeling. Additionally, nintedanib improved the overall health and lifespan of mice. However, pulmonary fibrosis and health deterioration of mice were not alleviated in the 12wPost group at 22 weeks post-radiation. These findings suggested that nintedanib should be administered at an early stage to exert beneficial effects on RIPF.

The exposure duration and radiation dose for both epithelial cells and fibroblasts were evaluated *in vitro*. Pulmonary cells and fibroblasts exhibited the highest radiation-induced intracellular alterations at 24 and 48 hours post-radiation, respectively. These findings indicate that the epithelial cell inflammatory response precedes the differentiation of fibroblasts, aligning with the results of prior research 10, 55. Following irradiation at a dosage of 8 Gy, the phosphorylation levels of the majority of inflammation-related proteins were upregulated in both epithelial cells and fibroblasts. Irradiation at a dose of 10 Gy downregulated the phosphorylation of some proteins, such as PI3K and p65. Therefore, higher radiation doses may cause cell death rather than regulating the inflammatory response or fibroblast differentiation. Thus, we use the irradiation at a dose of 8 Gy instead of 10 Gy for the further experiments.

Epithelial cells serve as the critical target cells in RILI 56. The inflammatory response of these epithelial cells is a vital effector process of pulmonary fibrosis 57. Several cytokines and chemokines from epithelial cells are activated in radiation-exposed lung tissue and are reported to promote the progression of RIPF 58. The PI3K/AKT signaling pathway holds significant significance in pro-inflammatory mediators expression and inflammatory cell recruitment, thereby contributing to the initiation and progression of lung inflammation 59. In this study, western blotting analysis revealed that nintedanib suppressed the radiation-induced upregulation of phosphorylated PI3K and AKT. The MAPK signaling pathway mediates intracellular signal transduction and regulates inflammatory responses 60. Meanwhile, the NF-κB signaling pathway serves as a mediator for the radiation-induced upregulation of pro-inflammatory cytokines 61. In this research, nintedanib alleviated the radiation-induced upregulation of p38 MAPK and ERK1/2 phosphorylation, as well as the NF-κB p65 phosphorylation. These data suggest that nintedanib downregulates inflammatory cytokine expression in radiation-treated epithelial cells by inhibiting the phosphorylation of PI3K/AKT and MAPK signaling pathway-related proteins.

The activation and differentiation of fibroblasts into myofibroblasts are critical for lung fibrosis development 62. Primarily, myofibroblasts arise from the differentiation of local tissue fibroblasts, which are characterized by the expression of α-SMA and enhanced production and secretion of ECM proteins 63. Therefore, the accumulation of myofibroblasts within pathological lesions serves as a hallmark of multiple diseases associated with fibrosis. Thus, the inhibition of fibroblast differentiation is a potential preventive strategy for lung fibrosis 64, 65. In this research, the administration of nintedanib substantially reduced inflammation and deposition of ECM in a murine model of RIPF. Moreover, the intervention of nintedanib downregulated the levels of α-SMA, CTGF and COL1A1. Thus, nintedanib exerts therapeutic effects on RIPF by suppressing the differentiation of fibroblast *in vivo*. Moreover, *in vitro* experiments demonstrated the inhibitory effect of nintedanib on radiation-induced fibroblast differentiation.

TGF-β1 is a key mediator of the differentiation of fibroblasts into pro-fibrotic myofibroblasts 64, 66. Injury or fibrotic stimulus promotes the release of TGF-β1 and the subsequent activation of the canonical Smad2/3 pathway 67-69. The elevation in TGF-β1 levels in both plasma and bronchoalveolar lavage fluid directly correlates with the severity of RIPF 70, 71. In this study, nintedanib downregulated the levels of TGF-β1 in the mouse serum and lung tissues after radiation exposure. Additionally, it inhibited the radiation-induced upregulation of phosphorylated Smad2/3. These findings demonstrate that the inhibitory impact of nintedanib on fibroblast differentiation is associated with its ability to alleviate the radiation-induced upregulation of TGF-β1 and the activation of the Smad signaling pathway *in vitro*. TGF-β1 can activate non-canonical signaling pathways involved in fibrosis, including MAPK, PI3K/AKT, and Rho-like GTPase signaling pathways 72-74. The development of pulmonary fibrosis was closely related to the interaction between TGF-β and PI3K/Akt 75. Furthermore, the activation of PI3K/Akt can activate the downstream targets such as mTOR. Targeting the PI3K/Akt/mTOR signaling cascade holds significant therapeutic potential in pulmonary fibrosis 76, 77. Our results revealed that nintedanib considerably reduced the radiation-induced upregulation of the expression of PI3K, AKT, and mTOR *in vitro*. Furthermore, it remarkably lowered α-SMA and COL1A1 levels in radiation-treated fibroblasts. These findings align with the hypothesis that nintedanib treatment can counteract the inhibition of fibroblast differentiation by suppressing the PI3K/Akt/mTOR signaling pathway.

However, there are some limitations to this study. Herein, we present evidence demonstrating the effectiveness of nintedanib in alleviating RIPF through targeting epithelial cells and fibroblasts. However, further investigation into the effects of nintedanib on other cell types, such as macrophages, neutrophils and T cells, is necessary. Moreover, our current investigation solely employed a single dose of radiation to establish the RIPF model, necessitating the evaluation of the efficacy of nintedanib under fractionated radiation doses, which better simulates clinical radiotherapy conditions. Thus, further extensive research is needed in this direction. Despite these limitations, our study proposed that nintedanib has promising potential to minimize RIPF by reducing epithelial cell inflammatory responses and inhibiting fibroblast-to-myofibroblast transition. These findings have important implications for prevention and treatment for RIPF and enrich understanding of the molecular mechanisms of RIPF, providing an experimental basis for the development of new drugs for the treatment of RIPF.

## Conclusions

This research demonstrated that the early treatment of nintedanib could suppress RIPF *in vitro* and* in vivo*. The mechanism underlying the therapeutic impact of nintedanib on RIPF was revealed to involve the inhibition of epithelial cell inflammatory responses and fibroblast differentiation and the suppression of the PI3K/AKT and MAPK pathways in epithelial cells and the PI3K/AKT/mTOR and TGF-β/Smad pathways in fibroblasts. These findings suggest the broad-spectrum therapeutic efficacy of nintedanib with multiple targets and encourage further clinical trials to determine the efficacy of nintedanib in patients with RIPF.

## Supplementary Material

Supplementary figures and table.
