# Supplementary material for: Nintedanib Mitigates Radiation-Induced Pulmonary Fibrosis by Suppressing Epithelial Cell Inflammatory Response and Inhibiting Fibroblast-to-Myofibroblast Transition
Source: Int J Biol Sci. 2024 Jun 11;20(9):3353–71. doi: 10.7150/ijbs.92620 (PMC11234214; doi:10.7150/ijbs.92620)
Supplement: Supplementary file 1 — Supplementary figures and table. [file ijbsv20p3353s1.pdf]

## Supplementary Material

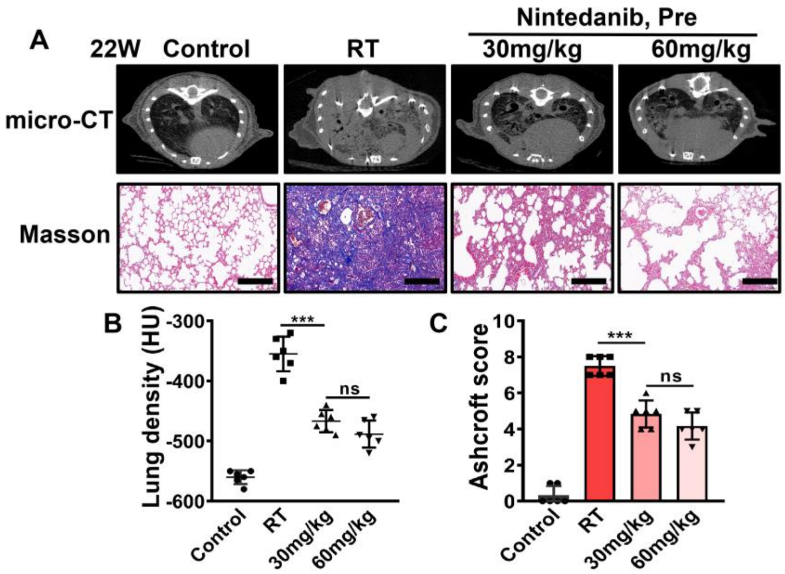


Figure S1. (A) Representative micro-CT images Masson's trichrome staining of lung samples from different treatment groups at week 22 post-radiation. (B) Assessment of lung density in micro-CT images. (C) Ashcroft score was used to determine the pulmonary fibrosis degree. $^{*}P < 0.05$, $^{**}P < 0.01$, $^{***}P < 0.001$, ns: not significant.

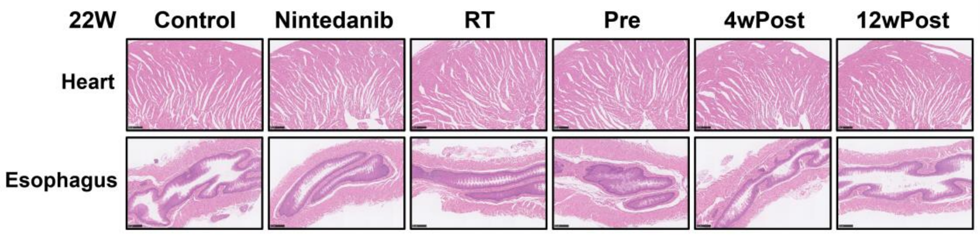


Figure S2. The results of hematoxylin and eosin (H&E) staining of heart and esophagus samples obtained at week 22 post-radiation (scale bar = 250 μm).

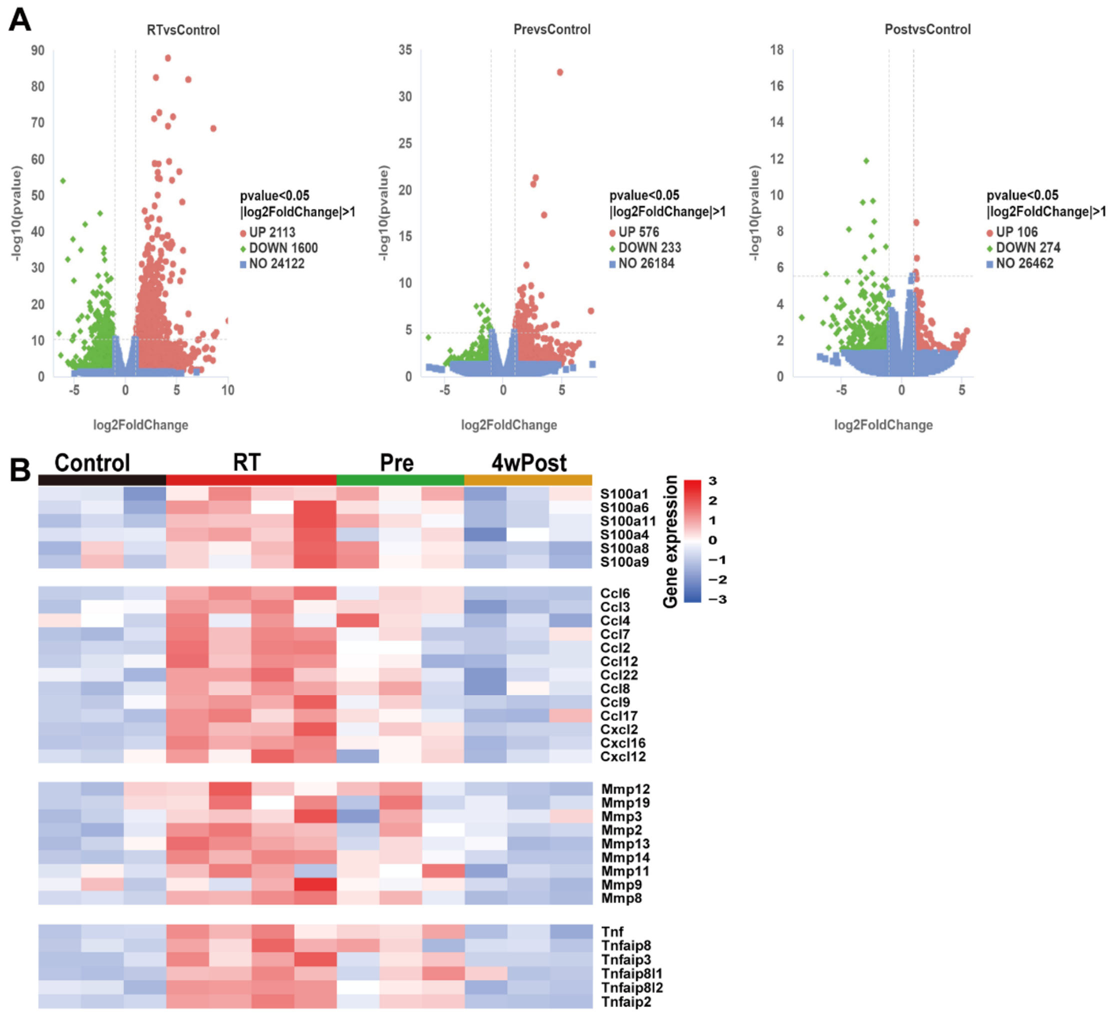


Figure S3. (A) Volcano plot of selected DEGs in the RT, Pre and 4wPost groups relative to control. (B) The expression levels of representative inflammation-related genes and ECM remodeling-related genes.

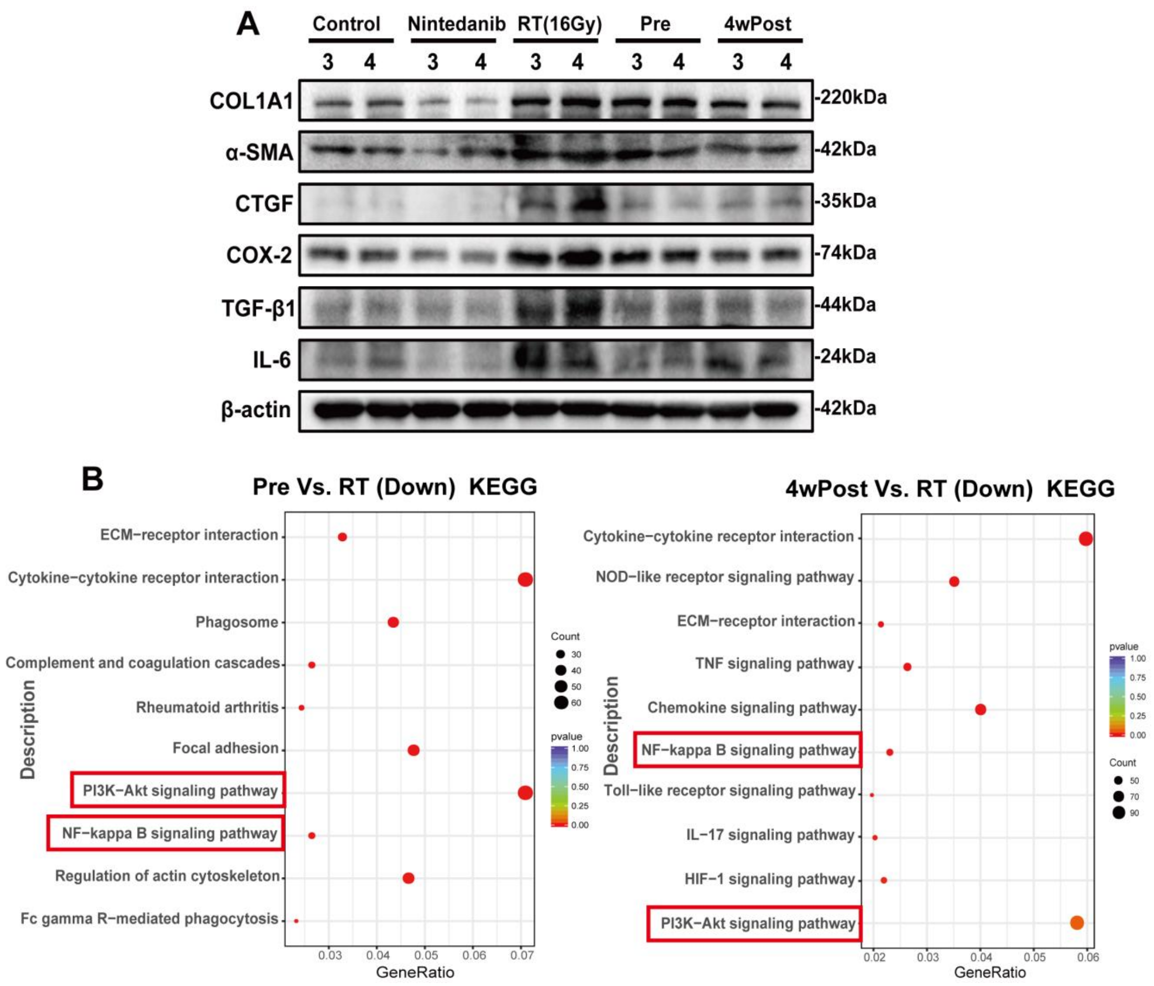


Figure S4. (A) Representative western blot images of COL1A1, α-SMA, CTGF, COX-2, TGF-β and IL-6 in lung tissue of mice. (B) Enriched KEGG dot plot from Pre vs. RT (down) groups and 4wPost vs. RT (down) groups. Noteworthy signaling pathways were marked with red rectangles.

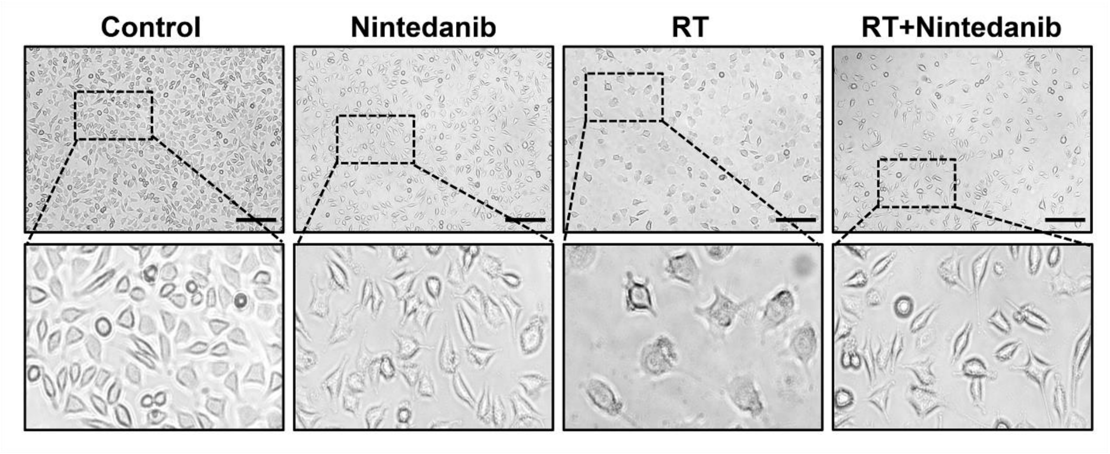


Figure S5. The morphological changes in L929 fibroblasts after different treatments. Scale bar = 200 μm.

Table S1. Union list of the top 100 inflammation-related GO terms for the DEGs from the RT vs. control groups, Pre vs. control groups, and 4wPost vs. control groups.

| GOID | Description | GeneRatio | pvalue |
|---|---|---|---|
| GO:0035456 | response to interferon-beta | 12/427 | 1.29E-09 |
| GO:0035458 | cellular response to interferon-beta | 11/427 | 1.46E-09 |
| GO:0071346 | cellular response to interferon-gamma | 12/427 | 9.57E-06 |
| GO:0034341 | response to interferon-gamma | 12/427 | 6.34E-05 |
| GO:0060330 | regulation of response to interferon-gamma | 4/427 | 3.23E-04 |
| GO:0060334 | regulation of interferon-gamma-mediated signaling pathway | 4/427 | 3.23E-04 |
| GO:0072593 | reactive oxygen species metabolic process | 16/427 | 4.93E-04 |
| GO:0060333 | interferon-gamma-mediated signaling pathway | 4/427 | 1.39E-03 |
| GO:0010634 | positive regulation of epithelial cell migration | 10/427 | 1.47E-03 |
| GO:0001961 | positive regulation of cytokine-mediated signaling pathway | 5/427 | 3.49E-03 |
| GO:0000305 | response to oxygen radical | 4/427 | 5.00E-03 |
| GO:0060760 | positive regulation of response to cytokine stimulus | 5/427 | 5.76E-03 |
| GO:0014909 | smooth muscle cell migration | 7/427 | 5.90E-03 |
| GO:0010595 | positive regulation of endothelial cell migration | 7/427 | 6.29E-03 |
| GO:0070665 | positive regulation of leukocyte proliferation | 9/427 | 7.82E-03 |
| GO:0034599 | cellular response to oxidative stress | 13/427 | 7.90E-03 |
| GO:0060326 | cell chemotaxis | 13/427 | 1.09E-02 |
| GO:0002443 | leukocyte mediated immunity | 63/999 | 5.46E-19 |
| GO:0030595 | leukocyte chemotaxis | 48/999 | 8.64E-19 |
| GO:0002250 | adaptive immune response | 67/999 | 1.48E-18 |
| GO:0060326 | cell chemotaxis | 56/999 | 1.86E-18 |
| GO:0050900 | leukocyte migration | 61/999 | 2.27E-18 |
| GO:0097529 | myeloid leukocyte migration | 45/999 | 3.75E-18 |
| GO:0002694 | regulation of leukocyte activation | 76/999 | 1.66E-16 |
| GO:0002274 | myeloid leukocyte activation | 44/999 | 3.50E-16 |
| GO:0002697 | regulation of immune effector process | 61/999 | 1.28E-15 |
| GO:0030593 | neutrophil chemotaxis | 29/999 | 1.38E-15 |
| GO:0097530 | granulocyte migration | 35/999 | 1.49E-15 |
| GO:0071621 | granulocyte chemotaxis | 32/999 | 2.81E-15 |
| GO:1990266 | neutrophil migration | 31/999 | 6.13E-15 |
| GO:0001819 | positive regulation of cytokine production | 63/999 | 1.23E-13 |
| GO:0002253 | activation of immune response | 57/999 | 2.00E-13 |
| GO:0019221 | cytokine-mediated signaling pathway | 56/999 | 2.48E-13 |
| GO:0006909 | phagocytosis | 39/999 | 2.89E-13 |
| GO:0002703 | regulation of leukocyte mediated immunity | 40/999 | 6.98E-13 |
| GO:0031349 | positive regulation of defense response | 52/999 | 9.71E-13 |
| GO:0002444 | myeloid leukocyte mediated immunity | 26/999 | 1.03E-12 |

| | | | |
|---|---|---|---|
| GO:0046651 | lymphocyte proliferation | 48/999 | 3.72E-12 |
| GO:0006959 | humoral immune response | 32/999 | 3.79E-12 |
| GO:0032943 | mononuclear cell proliferation | 48/999 | 4.90E-12 |
| GO:0050663 | cytokine secretion | 40/999 | 9.05E-12 |
| GO:0042110 | T cell activation | 64/999 | 1.07E-11 |
| GO:0050670 | regulation of lymphocyte proliferation | 41/999 | 1.08E-11 |
| GO:0032944 | regulation of mononuclear cell proliferation | 41/999 | 1.47E-11 |
| GO:0002696 | positive regulation of leukocyte activation | 49/999 | 1.49E-11 |
| GO:0050727 | regulation of inflammatory response | 49/999 | 1.69E-11 |
| GO:0070661 | leukocyte proliferation | 48/999 | 2.11E-11 |
| GO:0002449 | lymphocyte mediated immunity | 42/999 | 2.28E-11 |
| GO:0002526 | acute inflammatory response | 28/999 | 2.51E-11 |
| GO:0002764 | immune response-regulating signaling pathway | 48/999 | 3.50E-11 |
| GO:0070663 | regulation of leukocyte proliferation | 41/999 | 3.64E-11 |
| GO:0002275 | myeloid cell activation involved in immune response | 23/999 | 4.86E-11 |
| GO:0002886 | regulation of myeloid leukocyte mediated immunity | 19/999 | 5.18E-11 |
| GO:0051249 | regulation of lymphocyte activation | 57/999 | 6.03E-11 |
| GO:0002685 | regulation of leukocyte migration | 36/999 | 6.51E-11 |
| GO:0002690 | positive regulation of leukocyte chemotaxis | 24/999 | 6.70E-11 |
| GO:0002688 | regulation of leukocyte chemotaxis | 27/999 | 7.10E-11 |
| GO:0002819 | regulation of adaptive immune response | 32/999 | 7.79E-11 |
| GO:0045088 | regulation of innate immune response | 42/999 | 8.23E-11 |
| GO:0034612 | response to tumor necrosis factor | 34/999 | 1.14E-10 |
| GO:0002366 | leukocyte activation involved in immune response | 40/999 | 1.20E-10 |
| GO:0019724 | B cell mediated immunity | 27/999 | 1.40E-10 |
| GO:0002687 | positive regulation of leukocyte migration | 30/999 | 1.81E-10 |
| GO:0002263 | cell activation involved in immune response | 40/999 | 2.10E-10 |
| GO:0050764 | regulation of phagocytosis | 23/999 | 2.54E-10 |
| GO:0002521 | leukocyte differentiation | 66/999 | 3.03E-10 |
| GO:0034341 | response to interferon-gamma | 28/999 | 3.36E-10 |
| GO:0016064 | immunoglobulin mediated immune response | 26/999 | 4.85E-10 |
| GO:0045807 | positive regulation of endocytosis | 31/999 | 6.05E-10 |
| GO:0007159 | leukocyte cell-cell adhesion | 46/999 | 7.74E-10 |
| GO:0002757 | immune response-activating signal transduction | 44/999 | 7.84E-10 |
| GO:0050707 | regulation of cytokine secretion | 34/999 | 9.26E-10 |
| GO:0030100 | regulation of endocytosis | 40/999 | 1.53E-09 |
| GO:0002821 | positive regulation of adaptive immune response | 24/999 | 1.97E-09 |
| GO:0042098 | T cell proliferation | 35/999 | 2.51E-09 |
| GO:1903555 | regulation of tumor necrosis factor superfamily cytokine production | 28/999 | 2.68E-09 |
| GO:0002705 | positive regulation of leukocyte mediated immunity | 26/999 | 3.65E-09 |
| GO:0071706 | tumor necrosis factor superfamily cytokine production | 28/999 | 4.53E-09 |

| | | | |
|---|---|---|---|
| GO:0050766 | positive regulation of phagocytosis | 18/999 | 4.53E-09 |
| GO:0071674 | mononuclear cell migration | 19/999 | 6.33E-09 |
| GO:1903037 | regulation of leukocyte cell-cell adhesion | 41/999 | 6.44E-09 |
| GO:0006911 | phagocytosis, engulfment | 16/999 | 8.32E-09 |
| GO:0032680 | regulation of tumor necrosis factor production | 27/999 | 8.37E-09 |
| GO:0002446 | neutrophil mediated immunity | 13/999 | 1.00E-08 |
| GO:0050900 | leukocyte migration | 146/3441 | 1.19E-30 |
| GO:0050778 | positive regulation of immune response | 205/3441 | 5.97E-29 |
| GO:0060326 | cell chemotaxis | 122/3441 | 1.09E-24 |
| GO:0001819 | positive regulation of cytokine production | 164/3441 | 1.41E-24 |
| GO:0002274 | myeloid leukocyte activation | 97/3441 | 9.33E-24 |
| GO:0097529 | myeloid leukocyte migration | 91/3441 | 2.70E-22 |
| GO:0030595 | leukocyte chemotaxis | 96/3441 | 5.67E-22 |
| GO:0002253 | activation of immune response | 141/3441 | 1.19E-21 |
| GO:0031349 | positive regulation of defense response | 130/3441 | 1.33E-21 |
| GO:0030335 | positive regulation of cell migration | 184/3441 | 8.32E-21 |
| GO:0002250 | adaptive immune response | 143/3441 | 1.80E-20 |
| GO:0002685 | regulation of leukocyte migration | 91/3441 | 4.28E-20 |
| GO:0045088 | regulation of innate immune response | 108/3441 | 1.82E-19 |
| GO:0002443 | leukocyte mediated immunity | 128/3441 | 2.17E-19 |
| GO:0002764 | immune response-regulating signaling pathway | 123/3441 | 2.74E-19 |
| GO:0002521 | leukocyte differentiation | 183/3441 | 4.29E-19 |
| GO:0097530 | granulocyte migration | 69/3441 | 4.45E-19 |
| GO:0006909 | phagocytosis | 86/3441 | 1.87E-18 |
| GO:0002757 | immune response-activating signal transduction | 117/3441 | 2.09E-18 |
| GO:0050663 | cytokine secretion | 95/3441 | 2.12E-18 |
| GO:0050727 | regulation of inflammatory response | 120/3441 | 2.83E-17 |
| GO:0002687 | positive regulation of leukocyte migration | 71/3441 | 4.62E-17 |
| GO:0002694 | regulation of leukocyte activation | 169/3441 | 5.72E-17 |
| GO:0071216 | cellular response to biotic stimulus | 86/3441 | 6.29E-17 |
| GO:0019221 | cytokine-mediated signaling pathway | 130/3441 | 8.14E-17 |
| GO:0098542 | defense response to other organism | 153/3441 | 8.33E-17 |
| GO:0050707 | regulation of cytokine secretion | 84/3441 | 1.61E-16 |
| GO:0002460 | adaptive immune response based on somatic recombination of immune receptors built from immunoglobulin superfamily domains | 102/3441 | 2.38E-16 |
| GO:0032103 | positive regulation of response to external stimulus | 111/3441 | 3.13E-16 |
| GO:0009611 | response to wounding | 155/3441 | 3.40E-16 |
| GO:1990266 | neutrophil migration | 57/3441 | 3.64E-16 |
| GO:0042110 | T cell activation | 159/3441 | 7.36E-16 |
| GO:0032635 | interleukin-6 production | 67/3441 | 8.39E-16 |
| GO:0045089 | positive regulation of innate immune response | 88/3441 | 9.07E-16 |
| GO:0007159 | leukocyte cell-cell adhesion | 117/3441 | 1.20E-15 |
| GO:0032675 | regulation of interleukin-6 production | 64/3441 | 2.09E-15 |

| | | | |
|---|---|---|---|
| GO:0002688 | regulation of leukocyte chemotaxis | 58/3441 | 7.25E-15 |
| GO:0050715 | positive regulation of cytokine secretion | 64/3441 | 1.50E-14 |
| GO:0002697 | regulation of immune effector process | 126/3441 | 1.52E-14 |
| GO:0071621 | granulocyte chemotaxis | 56/3441 | 1.85E-14 |
| GO:0002366 | leukocyte activation involved in immune response | 93/3441 | 2.68E-14 |
| GO:0002263 | cell activation involved in immune response | 94/3441 | 3.29E-14 |
| GO:0070661 | leukocyte proliferation | 111/3441 | 4.90E-14 |
| GO:0032943 | mononuclear cell proliferation | 107/3441 | 8.72E-14 |
| GO:0046651 | lymphocyte proliferation | 106/3441 | 1.33E-13 |
| GO:0002224 | toll-like receptor signaling pathway | 50/3441 | 1.75E-13 |
| GO:0002690 | positive regulation of leukocyte chemotaxis | 48/3441 | 2.49E-13 |
| GO:0030198 | extracellular matrix organization | 88/3441 | 3.41E-13 |
| GO:1903555 | regulation of tumor necrosis factor superfamily cytokine production | 64/3441 | 3.53E-13 |
| GO:0002573 | myeloid leukocyte differentiation | 84/3441 | 3.99E-13 |
| GO:0042060 | wound healing | 119/3441 | 4.35E-13 |
| GO:0051249 | regulation of lymphocyte activation | 136/3441 | 7.27E-13 |
| GO:0043410 | positive regulation of MAPK cascade | 164/3441 | 1.11E-12 |
| GO:0071706 | tumor necrosis factor superfamily cytokine production | 64/3441 | 1.23E-12 |
| GO:0002699 | positive regulation of immune effector process | 81/3441 | 1.52E-12 |
| GO:0032680 | regulation of tumor necrosis factor production | 62/3441 | 2.08E-12 |
| GO:0002218 | activation of innate immune response | 64/3441 | 2.75E-12 |
| GO:0002449 | lymphocyte mediated immunity | 90/3441 | 3.00E-12 |
| GO:1902105 | regulation of leukocyte differentiation | 101/3441 | 4.18E-12 |
| GO:0032612 | interleukin-1 production | 43/3441 | 4.50E-12 |
| GO:0032640 | tumor necrosis factor production | 62/3441 | 4.69E-12 |
| GO:0030593 | neutrophil chemotaxis | 45/3441 | 5.49E-12 |
| GO:0032611 | interleukin-1 beta production | 37/3441 | 1.08E-11 |
| GO:0002758 | innate immune response-activating signal transduction | 57/3441 | 2.15E-11 |
| GO:0002703 | regulation of leukocyte mediated immunity | 77/3441 | 3.13E-11 |
| GO:0001776 | leukocyte homeostasis | 51/3441 | 4.33E-11 |
| GO:0034612 | response to tumor necrosis factor | 70/3441 | 5.49E-11 |
| GO:0032609 | interferon-gamma production | 52/3441 | 6.43E-11 |
| GO:0032649 | regulation of interferon-gamma production | 48/3441 | 7.28E-11 |
| GO:0072593 | reactive oxygen species metabolic process | 92/3441 | 7.71E-11 |
| GO:0050729 | positive regulation of inflammatory response | 51/3441 | 1.03E-10 |
| GO:1903037 | regulation of leukocyte cell-cell adhesion | 96/3441 | 1.12E-10 |
